# Too dim, too bright, and just right: Systems analysis of the *Chlamydomonas* diurnal program under limiting and excess light

**DOI:** 10.1093/plcell/koaf086

**Published:** 2025-04-19

**Authors:** Sunnyjoy Dupuis, Valle Ojeda, Sean D Gallaher, Samuel O Purvine, Anne G Glaesener, Raquel Ponce, Carrie D Nicora, Kent Bloodsworth, Mary S Lipton, Krishna K Niyogi, Masakazu Iwai, Sabeeha S Merchant

**Affiliations:** Department of Plant and Microbial Biology, University of California, Berkeley, CA 94720, USA; California Institute for Quantitative Biosciences, University of California, Berkeley, CA 94720, USA; California Institute for Quantitative Biosciences, University of California, Berkeley, CA 94720, USA; California Institute for Quantitative Biosciences, University of California, Berkeley, CA 94720, USA; Earth and Biological Sciences Division, Pacific Northwest National Laboratory, Richland, WA 99352, USA; California Institute for Quantitative Biosciences, University of California, Berkeley, CA 94720, USA; Department of Plant and Microbial Biology, University of California, Berkeley, CA 94720, USA; Howard Hughes Medical Institute, University of California, Berkeley, CA 94720, USA; Earth and Biological Sciences Division, Pacific Northwest National Laboratory, Richland, WA 99352, USA; Earth and Biological Sciences Division, Pacific Northwest National Laboratory, Richland, WA 99352, USA; Earth and Biological Sciences Division, Pacific Northwest National Laboratory, Richland, WA 99352, USA; Department of Plant and Microbial Biology, University of California, Berkeley, CA 94720, USA; Howard Hughes Medical Institute, University of California, Berkeley, CA 94720, USA; Molecular Biophysics and Integrated Bioimaging Division, Lawrence Berkeley National Laboratory, Berkeley, CA 94720, USA; Department of Plant and Microbial Biology, University of California, Berkeley, CA 94720, USA; Molecular Biophysics and Integrated Bioimaging Division, Lawrence Berkeley National Laboratory, Berkeley, CA 94720, USA; Department of Plant and Microbial Biology, University of California, Berkeley, CA 94720, USA; California Institute for Quantitative Biosciences, University of California, Berkeley, CA 94720, USA; Environmental Genomics and Systems Biology Division, Lawrence Berkeley National Laboratory, Berkeley, CA 94720, USA; Department of Molecular and Cell Biology, University of California, Berkeley, CA 94720, USA

## Abstract

Photosynthetic organisms coordinate their metabolism and growth with diurnal light, which can range in intensity from limiting to excessive. Little is known about how light intensity impacts the diurnal program in *Chlamydomonas reinhardtii*, or how diurnal rhythms in gene expression and metabolism shape photoprotective responses at different times of day. To address these questions, we performed a systems analysis of synchronized *Chlamydomonas* populations acclimated to low, moderate, and high diurnal light. Transcriptomic and proteomic data revealed that the *Chlamydomonas* rhythmic gene expression program is resilient to limiting and excess light: genome-wide, waves of transcripts, and proteins peak at the same times in populations acclimated to stressful light intensities as in populations acclimated to moderate light. Yet, diurnal photoacclimation gives rise to hundreds of gene expression changes, even at night. Time course measurements of photosynthetic efficiency and pigments responsive to excess light showed that high light-acclimated cells partially overcome photodamage in the latter half of the day prior to cell division. Although gene expression and photodamage are dynamic over the diurnal cycle, *Chlamydomonas* populations acclimated to low and high diurnal light maintain altered photosystem abundance, thylakoid architecture, and non-photochemical quenching capacity through the night phase. This suggests that cells remember or anticipate the light intensities that they have typically encountered during the day. The integrated data constitute an excellent resource for understanding photoacclimation in eukaryotes under environmentally relevant conditions.

## Teaser

*Chlamydomonas* populations acclimate to low and high diurnal light and maintain altered photosystem abundance, thylakoid architecture, and non-photochemical quenching capacity during the night.

## Introduction

Photosynthetic organisms have evolved under a highly dynamic light environment, where the intensity of solar irradiance varies over several orders of magnitude each day (Long et al. 2022). The periodic availability of sunlight over diurnal cycles restricts photosynthetic activity to the daytime, yielding daily rhythms in cellular growth and metabolic activity (Dodd et al. 2005; Oravec and Greenham 2022; Gendron and Staiger 2023). Over each day period, incident light intensity can range from limiting to excess. Under excess light, the rate of light absorption exceeds the rate of photosynthetic electron transfer, leading to production of reactive chemical species that can cause photooxidative damage. Accordingly, these organisms have evolved sophisticated mechanisms to sense light intensity, adjust light-harvesting capacities, dissipate excess absorbed energy, and repair photodamage (Croce and van Amerongen 2020; Bassi and Dall’Osto 2021; Iwai et al. 2024). In photosynthetic eukaryotes, these mechanisms are coordinated across cellular compartments through retrograde signaling from the chloroplast and mitochondria to the nucleus (Chan et al. 2016).

The unicellular, eukaryotic green alga *Chlamydomonas reinhardtii* (*Chlamydomonas* hereafter) is a classic model organism for studying photosynthesis and its regulation (Dupuis and Merchant 2023). As a unicellular organism, it avoids the tissue- and developmental-stage-specific heterogeneities that complicate studies in land plants are avoided. Yet, having a shared evolutionary history with land plants, *Chlamydomonas* harbors conserved photosynthetic machinery, regulatory pathways, and photoprotective mechanisms. When the amount of light absorbed by chlorophyll (Chl) exceeds the capacity for photochemical quenching by charge separation, non-photochemical quenching (NPQ) mechanisms are induced that dissipate energy as heat. These mechanisms operate over both short and long timescales (Erickson et al. 2015; Iwai et al. 2023). The most rapidly reversible NPQ, called energy-dependent quenching or qE, is performed by energy-dissipative light-harvesting complex (LHC) proteins (e.g. LHCSRs) that accumulate in thylakoid membranes alongside the photosynthetic apparatus in high light (HL) (Peers et al. 2009; Allorent et al. 2016; Petroutsos et al. 2016; Redekop et al. 2022; Ruiz-Sola et al. 2023). In addition, a slowly reversible NPQ, called zeaxanthin-dependent quenching or qZ, occurs when high rates of linear electron transfer from photosystem II (PSII) to photosystem I (PSI) increase the proton gradient across the thylakoid membrane (ΔpH), activating violaxanthin de-epoxidase (CVDE1) which converts violaxanthin (Vio) to zeaxanthin (Zea), an energy-dissipative carotenoid (Niyogi et al. 1997; Li et al. 2016; Troiano et al. 2021). Another slowly reversible NPQ, called state-transition-dependent quenching or qT, occurs when the high reduction state of the plastoquinone pool activates the STT7 kinase, which phosphorylates the LHC proteins of PSII (LHCII) (Depège et al. 2003). Phosphorylated LHCII trimers associate with PSI in State 2, thereby redistributing excitation energy between the photosystems (Allen et al. 1981; Depège et al. 2003). Upon prolonged HL exposure, photoinhibition itself can cause quenching in the PSII reaction center, known as qI, the most slowly reversible NPQ mechanism (Nawrocki et al. 2021).

In addition to NPQ, *Chlamydomonas* can acclimate to prolonged HL by decreasing Chl content and antenna size to reduce the excitation energy pressure on the photosystems, a process termed photoacclimation (Neale and Melis 1986; Anderson et al. 1988, 1995; Falkowski and LaRoche 1991; Durnford and Falkowski 1997; Shapira et al. 1997; Huner et al. 1998; Teramoto et al. 2002; Durnford et al. 2003; Arend et al. 2023). Most studies on photoacclimation in *Chlamydomonas* have used cultures maintained in continuous light to maximize growth rate or cultures shifted from one continuous light intensity to another (Neale and Melis 1986; Shapira et al. 1997; Teramoto et al. 2002; Durnford et al. 2003; Im et al. 2003; Petroutsos et al. 2011; Bonente et al. 2012; Mettler et al. 2014; Polukhina et al. 2016; Meagher et al. 2021; Redekop et al. 2022). However, in natural environments, photoacclimation occurs in the context of the diurnal light cycle, over which gene expression, metabolism, and physiology are coordinated with the time of day. Cell growth (G1 phase of the cell cycle) is restricted to the light phase. After a full day of growth, *Chlamydomonas* cells replicate their genomes (S phase) and undergo mitotic divisions (M phase) to produce daughter cells of equal sizes (Umen and Liu 2023). As a result, *Chlamydomonas* populations synchronize when grown under repeated diurnal cycles in the laboratory.

With millions of cells each at the same stage of the cell cycle, synchronous populations produce high signal-to-noise for measuring changes in gene expression and metabolism that would be obscured in batch cultures. Synchronous *Chlamydomonas* populations have been a workhorse for studying the cell cycle (Cross and Umen 2015). They have also revealed that metabolic pathways and the biogenesis of various cellular structures (e.g. photosynthetic and respiratory complexes, cilia, ribosomes) are segregated to specific times of day to optimize resource allocation over the diurnal cycle. Our previous work on synchronous populations grown under moderate diurnal light (ML) revealed that this diurnal program is achieved through rhythmic accumulation of ∼85% of nuclear and organellar transcripts (Strenkert et al. 2019). Interestingly, *LHCSRs* were among these rhythmically expressed genes, even at low light (LL), suggesting that the photoprotective mechanisms that *Chlamydomonas* engages could also be dynamic over the diurnal cycle. Only a few studies to date have explored this hypothesis. Nawrocki et al. (2020) found that under sinusoidal HL illumination, NPQ capacity remained high over time, which was attributed to the maintenance of LHCSR proteins during light and dark phases (Nawrocki et al. 2020). Using a similar sinusoidal light regime, van den Berg and Croce (2022) found that carotenoid composition varied significantly over the diurnal cycle in *Chlamydomonas* (van den Berg and Croce 2022).

Apart from these findings, it is largely unclear how diurnal rhythms in gene expression and metabolism influence photoprotection, and conversely, how the intensity of light may impact *Chlamydomonas*' diurnal program. As diurnal illumination is a fundamental characteristic of our planet, addressing these questions is critical to our understanding of photosynthetic organisms, their capacity to acclimate to changing environments, and our ability to engineer them for improved productivity. In previous work, we documented the diurnal program under 200 µmol photons m^−2^ s^−1^ of photosynthetically active radiation (light that is absorbed by Chl) (Strenkert et al. 2019). Now, we ask how that program is affected by lower energy input (LL, 50 µmol photons m^−2^ s^−1^), which limits growth, or very high energy input (HL, 1,000 µmol photons m^−2^ s^−1^), which may damage the photosynthetic apparatus. We also determine how the diurnal program shapes the response to light stress at different times of day and night. We present a comprehensive view of gene expression, photosynthesis, metabolism, and chloroplast morphology at five timepoints across the day and night that integrates transcriptomics, proteomics, lipidomics, pigments, bioimaging, and biophysical data. We find that diurnal rhythms in gene expression are resilient to limiting and excess light, but that diurnal photoacclimation gives rise to hundreds of gene expression changes, even at night. In addition, we show that HL-acclimated cells recover their photosynthetic efficiency and reduce their Zea content in the latter half of the day even when light intensity remains high. Although gene expression and photodamage are highly dynamic, we find that the acclimated populations maintain altered thylakoid architecture and photosystem abundance into the night phase. Retention of acclimatory phenotypes through the night may allow *Chlamydomonas* to prepare for the light intensities that it typically encounters during the day.

## Results

### The *Chlamydomonas* cell cycle is coordinated with the time of day even under challenging light intensities

**Figure 1.**
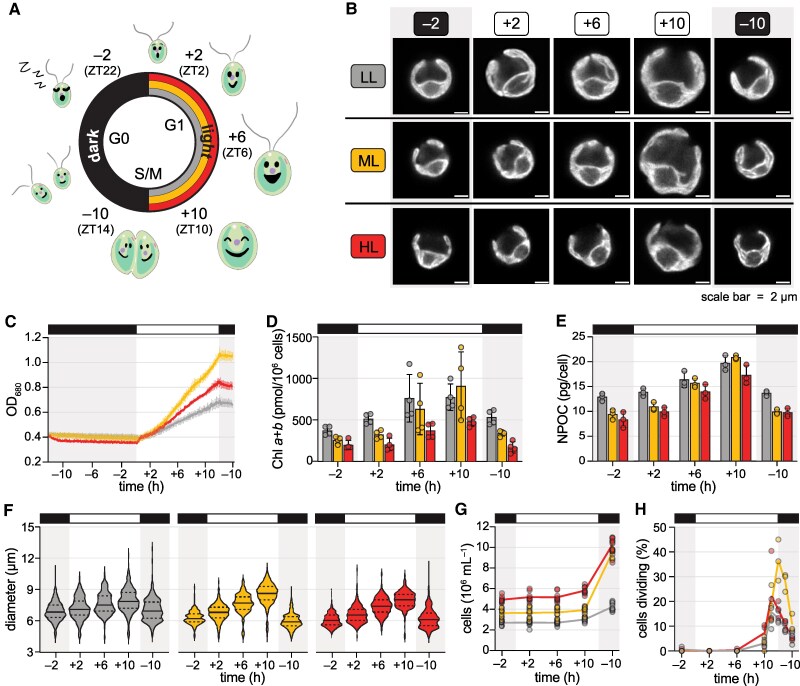
The *Chlamydomonas* cell cycle is coordinated with the time of day even under challenging light intensities. Growth of *Chlamydomonas* populations acclimated to diurnal low light (LL, grey), moderate light (ML, yellow), and high light (HL, red). **A)** Schematic of *Chlamydomonas* populations synchronized under 12-h-dark/12-h-light cycles of LL (grey), ML (yellow), and HL (red). Timepoints are numbered relative to the dark-to-light transition, and the corresponding Zeitgeber Time (ZT) is shown in parentheses. **B)** Representative live-cell Chl fluorescence images of the 15 cellular states assayed in this study: three photoacclimated populations (LL-acclimated, ML-acclimated, and HL-acclimated, rows) at five timepoints across the diurnal cycle (−2, +2, +6, +10, −10, columns). **C)** Optical density (OD_680_) of each culture was set to 0.4 at the beginning of the dark phase and continuously monitored over the 26 h experiments. Data are represented as the mean of at least 9 experimental replicates (*n* ≥ 9) with error bars representing the standard deviation from the mean. **D)** Cellular Chl content for 4 experimental replicates (*n* = 4). Error bars represent the standard deviation from the mean. **E)** Cellular organic carbon content, estimated as nonpurgeable organic carbon (NPOC) (*n* = 3). Error bars represent the standard deviation from the mean. **F)** Cell diameter measured by microscopy for 100 cells from each of three experimental replicates (*n* = 300); solid lines represent the median and dashed lines the quartiles. **G)** Cell density measured by a particle counter for 9 experimental replicates (*n* = 9). **H)** Approximate percent of cells dividing in the population at each time, as estimated by microscopy for three experimental replicates (*n* = 3). See also Supplementary Data Sets 8 and 9.

To understand how the quantity of photosynthetically active radiation influences the *Chlamydomonas* diurnal program, we acclimated synchronous populations to day–night cycles of one of three light intensities and then performed analytical measurements over a diurnal cycle time course. We used culture conditions previously established to maximize population synchrony (Strenkert et al. 2019). Cells were grown photoautotrophically on air levels of CO_2_ in turbidostat photobioreactors with red and blue LEDs (representing peaks of Chl absorption) under 12-h-light/12-h-dark cycles of warm days (28 °C) and cool nights (18 °C) as previously described, but the quantity of light each day was set to either LL, ML, or HL (∼50, ∼200, or ∼1,000 µmol photons m^−2^ s^−1^, respectively). The ML condition, which produces an exquisite level of synchrony in which cells divide once per diurnal cycle upon nightfall, has been studied extensively and served as our control (Strenkert et al. 2019). Populations were maintained in these conditions for >2 weeks prior to each experiment. Since the LL condition limited growth, these populations were first synchronized in diurnal ML for 1 week and then acclimated to diurnal LL for >1 week. We assayed physiology and gene expression of the acclimated populations at five timepoints, numbered relative to the dark-to-light transition: two hours before the light phase (−2), two, six, and ten hours into the light phase (+2, +6, +10), and two hours into the dark phase, which is ten hours before the next light phase (−10) (Fig. 1A). Thus, with three photoacclimated populations and five timepoints, we compared a total of 15 cellular states (Fig. 1B).

The acclimation conditions yielded excellent reproducibility of culture growth across independent replicate experiments performed months apart (Fig. 1C, mean relative standard deviation = 4.14%). Regardless of light intensity, growth was restricted to the light phase. The LL-acclimated population grew the most slowly and reached the lowest final density (Tukey's Honestly Significant Difference (HSD) tests, *p-adj.* < 0.0005), indicating that light energy input limits growth in LL. The HL-acclimated population also reached a lower density than the ML population did (Tukey's HSD test, *p-adj.* = 1.06 × 10^–11^), indicating that this light intensity is also suboptimal, most likely as a consequence of photoinhibition. As expected, LL-acclimated cells often had more Chl compared to HL-acclimated cells (Fig. 1D). Interestingly, this difference was maintained even after 10 h of dark (−2) (Games-Howell test, *p-adj.* = 0.002).

LL-acclimated cells also had a higher organic carbon content than did the ML and HL populations at the −2, +2, and −10 timepoints (Fig. 1E, Tukey's HSD tests, *p-adj.* < 0.05). This reflected a significant increase in the average size of LL cells at these timepoints (Fig. 1F, Dunn's tests, *p-adj* < 0.0005). Cell division was coordinated with the light-to-dark transition in all three populations: large cells began dividing at the end of the day, resulting in a decrease in cell diameter and a concomitant increase in cell density (Fig. 1, F to H). The number of cells in the ML and HL populations increased over two-fold on average, whereas the LL population did not quite double (mean fold-change = 1.58) (Fig. 1G). Thus, not all cells in the LL population passed the commitment point of the cell cycle each day, leaving more cells with large diameters. Nevertheless, for the cells that did divide, the division occurred synchronously during the light-to-dark transition (Fig. 1H), enabling greater signal-to-noise for measurements of gene expression and physiology relative to asynchronous populations grown under continuous light.

### The rhythmic diurnal gene expression program is maintained under diurnal LL and HL

**Figure 2.**
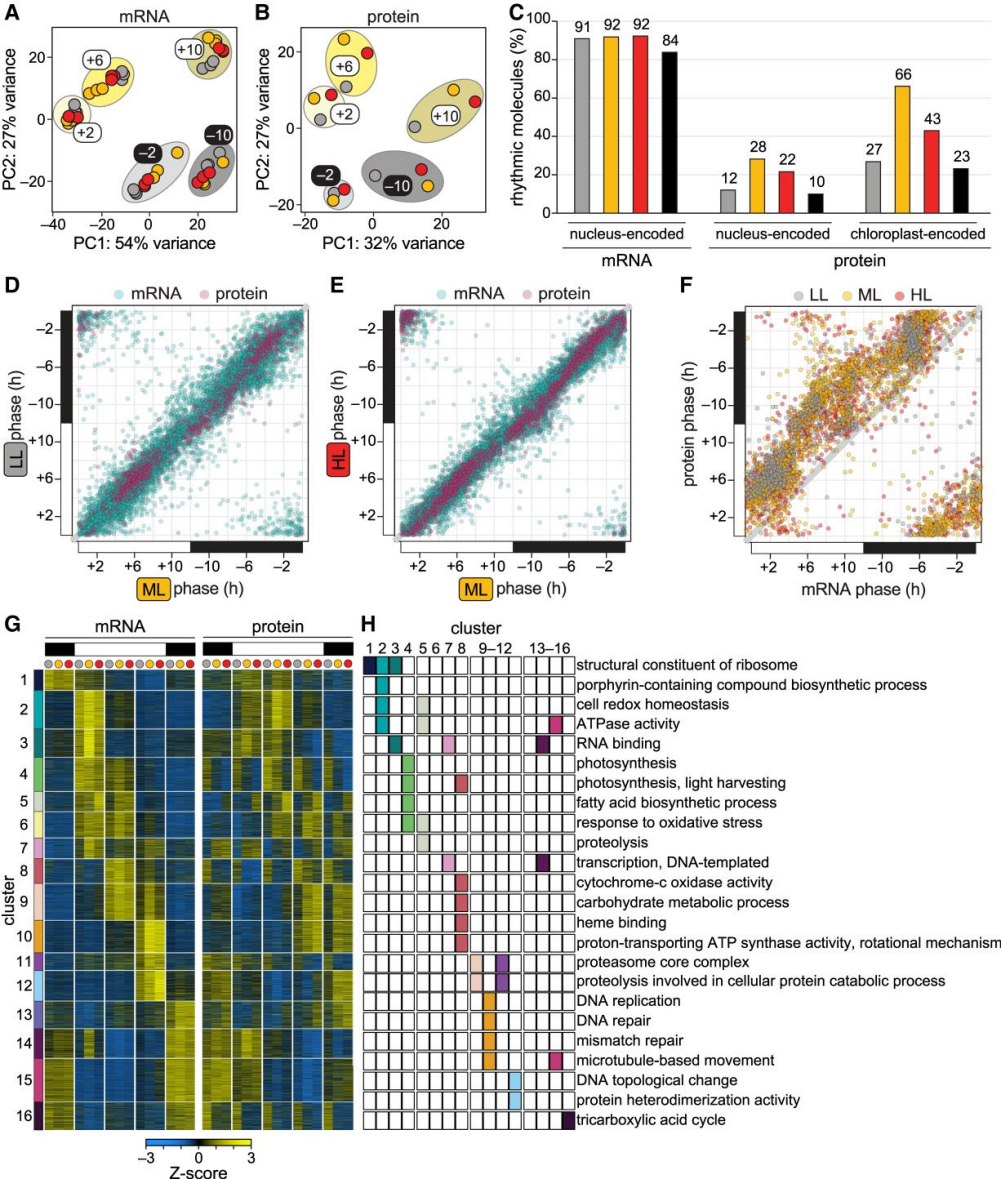
The rhythmic diurnal gene expression program is maintained under diurnal LL and HL. Gene expression in *Chlamydomonas* populations acclimated to diurnal low light (LL, grey), moderate light (ML, yellow), and high light (HL, red). **A)** PCA of mRNA abundances (FPKMs) genome-wide in three experimental replicates (*n* = 3); ellipses demonstrate grouping by time of day. **B)** PCA of mean protein abundances (MASIC values) genome-wide across three experimental replicates (*n* = 3); ellipses demonstrate grouping by time of day. See Methods section for more details. **C)** Proportion of detected molecules (nucleus- and chloroplast-encoded mRNAs and proteins) that exhibit a significant diurnal rhythm in abundance in diurnal LL (grey), ML (yellow) and HL (red) and in all three light intensities (black). Rhythmicity was assessed with a Cosinor algorithm in the R package *DiscoRhythm*. Expression patterns that matched an oscillation with a 24 h period with a *q*-value < 0.05 were defined as a significant diurnal rhythm. See Methods section for more details. No significant diurnal rhythms were detected for the 5 mitochondria-encoded proteins captured by TMT proteomics. **D)** Correlation between the phase of rhythmic gene expression (the time at which abundance peaks) in populations acclimated to diurnal LL and ML for mRNAs (blue) and proteins (purple) that had a significant diurnal rhythm in both photoacclimated populations (*q* < 0.05). **E)** Correlation between the phase of rhythmic gene expression in populations acclimated to diurnal HL and ML for mRNAs (blue) and proteins (purple) that had a significant diurnal rhythm in both photoacclimated populations (*q* < 0.05). **F)** Correlation between the phase of expression for mRNAs and their cognate proteins that had a significant diurnal rhythm (*q* < 0.05) in populations acclimated to LL, ML, and HL; grey line represents a 1:1 correlation. **G)** mRNA and protein levels for 9,821 genes grouped into 16 clusters by *k*-means clustering of their normalized expression patterns. Normalized values are the Z-score of the mean mRNA abundance (FPKM) and the Z-score of the mean protein abundance (MASIC value) of three experimental replicates (*n* = 3). **H)** Selected GO terms significantly enriched in the 16 clusters in Fig. 2G (*p-adj*. < 0.05). The number of genes, gene ratio, and *p-adj*. of enrichment are viewable in Supplementary Fig. S3. The full list of enriched GO terms is available as Supplementary Data Set 4. See also Supplementary Figs. S1 to S3, and Supplementary Data Sets 2 to 4.

To understand how acclimation to different light intensities impacts *Chlamydomonas*' rhythmic gene expression program, we performed RNA-Seq and tandem mass tag (TMT) proteomics (Supplementary Data Sets 1 and 2). We detected 16,735 nucleus-encoded mRNAs (95%) (Supplementary Fig. S1A), 10,011 nucleus-encoded proteins (56.9%), 59 chloroplast-encoded proteins (81.9%), and 5 mitochondria-encoded proteins (62.5%) (Supplementary Fig. S1B). Genome-wide, mRNA and protein abundances appeared to oscillate with a 24 h period over the diurnal cycle, and light intensity seemed to have little effect on the timing of accumulation. Principal component analysis (PCA) of the RNA-Seq data confirmed that most of the variation in the transcriptome is explained by time of day, rather than by light intensity (Fig. 2A). It also confirmed tight reproducibility across experimental replicates collected weeks apart, as is demonstrated by the high pairwise Pearson correlation coefficients (*r*) between replicates of each of the 15 cellular states (Supplementary Fig. S2A, *r* ≥ 0.98). Experimental replicates of the proteome were also highly correlated (pairwise *r* > 0.91), despite having been run in three randomized sample plexes which, expectedly, had a large influence on protein detection (Supplementary Figs. S2, A and B). PCA of the proteomics data in each of the three sample plexes and in the averaged data showed that time of day also drives the variation in protein abundances (Fig. 2B and Supplementary Figs. S2, C to E). For both the transcriptome and the proteome, the gene expression data are positioned into a clock-like pattern by the first two principal components, which together make up 81% of the variation in mRNA abundances and 59% of the variation in protein abundances.

Next, we used the R package *DiscoRhythm* to test for rhythmicity in the gene expression data (Carlucci et al. 2020). We employed *DiscoRhythm's* cosinor algorithm to determine which genes were expressed with a significant diurnal periodicity (24-h period, *q* < 0.05) in the three populations and to estimate the phase of these rhythms (the time at which expression peaks) (Cornelissen 2014; Carlucci et al. 2020) (Supplementary Data Set 3). We found that roughly 91% of nucleus-encoded transcripts exhibited a significant diurnal oscillation in the populations, whereas significant diurnal rhythms were only detected for 12% to 28% of nucleus-encoded proteins and 27% to 66% of chloroplast-encoded proteins depending on the diurnal light intensity (Fig. 2C, *q* < 0.05). A similar study that instead used data-independent acquisition proteomics in the marine alga *Ostreococcus* also found decreased rhythmicity of the proteome relative to the transcriptome over diurnal cycles: 80% of transcripts and 55% of proteins exhibited a diurnal rhythm (Romero-Losada et al. 2025). As both *k*-means clustering and PCA suggested that a large proportion of the *Chlamydomonas* proteome was rhythmically expressed (Supplementary Figs. S1B and S2, C to E), *DiscoRhythm* may underestimate rhythmicity in TMT proteomics data due to the compressed dynamic range.

For rhythmic mRNAs and proteins alike, the phase of expression in the LL and HL populations correlated well with the phase in the ML control population (Fig. 2, D and E, majority of points fall near the 1:1 line). Thus, the timing of gene expression is largely maintained regardless of the intensity of diurnal light in *Chlamydomonas*, demonstrating that the alga's diurnal program is robust and resilient to limiting and excess light. As predicted by the central dogma of biology (DNA to RNA to protein), we found that the phase for rhythmically expressed proteins often fell 2-to-8 h after the phase for the cognate mRNA (Fig. 2F).

Both mRNA and protein products were detected from 9,821 genes, and these genes were grouped into clusters based on their expression patterns (Fig. 2G). Of the 16 clusters, 14 were significantly enriched for gene ontology (GO) terms (hypergeometric tests, *p-adj.* < 0.05), revealing that the expression of genes encoding related functions was gated to specific times of day as reported previously (Zones et al. 2015; Strenkert et al. 2019) and hinting at light-responsive changes in cellular physiology (Fig. 2H and Supplementary Fig. S3, Supplementary Data Set 4). For example, Cluster 4 genes—enriched for “photosynthesis, light harvesting,” “fatty acid biosynthesis,” and “response to oxidative stress” terms—peaked in mRNA abundances early and midday (+2 and +6), and the corresponding proteins were more abundant in the LL population at all timepoints. Cluster 5 mRNAs accumulated similarly to those in Cluster 4, but proteins in this cluster were instead more abundant in the HL population, especially at midday (+6). This cluster was also significantly enriched with “response to oxidative stress” in addition to proteolysis-related terms, suggesting that the HL population may experience photooxidative damage at midday. Cluster 10, enriched for DNA replication and repair terms, was most highly expressed at the end of the day (+10) when the cells were in S and M phase of the cell cycle. The magnitude of expression of these genes was reduced in the LL population, in line with the observation that fewer LL cells divided upon nightfall (Fig. 1, G and H).

Taken together, our results reveal that the *Chlamydomonas* diurnal program is robust and resilient to light stress and limitation. Waves of gene expression peak at the same times of day to gate metabolic pathways and cellular processes to particular phases of the diurnal cycle, even in the face of stress.

### Hundreds of genes are differentially expressed in populations acclimated to diurnal LL and HL, even during the night

**Figure 3.**
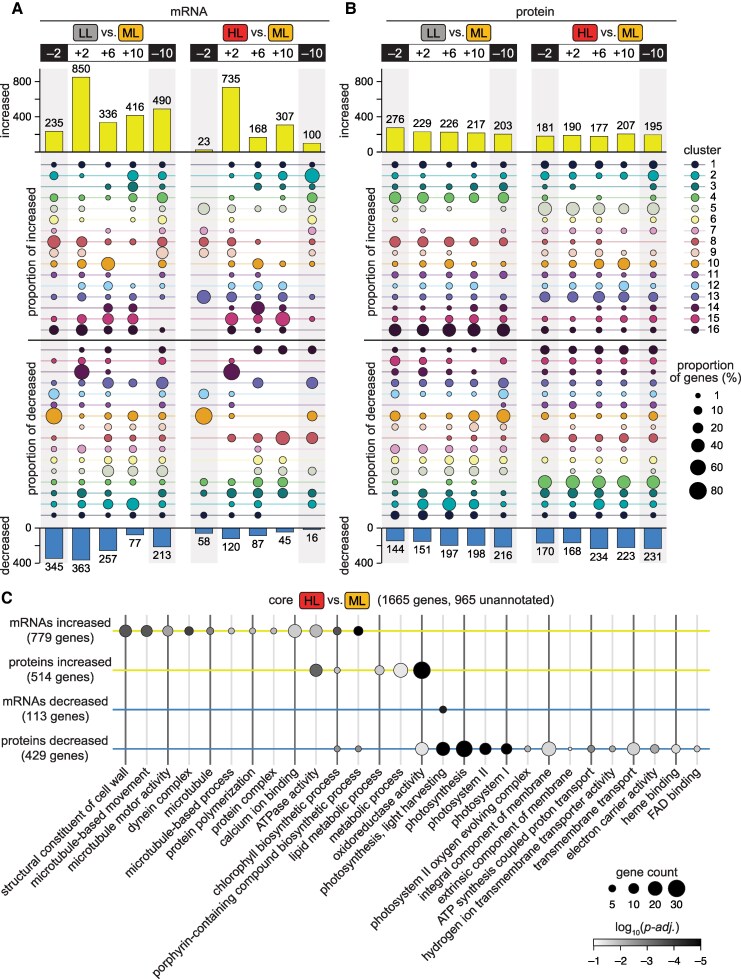
Hundreds of genes are differentially expressed in populations acclimated to diurnal low light (LL) and high light (HL) at all timepoints, and comparison reveals HL-specific changes related to motility and metabolism. **A)** The number of mRNAs significantly increased (top) and decreased (bottom) in abundance compared to the control population (ML, moderate light-acclimated) at each timepoint, and the proportion of those mRNAs that belong to each cluster in Fig. 2G (relative to those that belong to any cluster) represented as dot size (center); the key at right is scaled by the proportion of total genes in each cluster for comparison. See Methods for details about differential expression analysis. **B)** Significant differences in protein abundance represented as in **(A)**. **C)** GO term enrichment in the “core HL-responsive” gene expression changes. Core HL-responsive mRNAs and proteins are defined as either significantly increased in HL and unchanged or decreased in LL (top two rows, yellow), or significantly decreased in HL and unchanged or increased in LL (bottom two rows, blue). Dot size indicates the number of genes in each list represented by the GO term, and the shading indicates the log_10_*p-adj*. 965 of the 1,665 genes with an HL-specific change in expression do not have an associated gene symbol, indicating that they are of unknown or unverified function. See also Supplementary Figs. S4 to S6, and Supplementary Data Sets 5 and 6.

Although the diurnal program was largely maintained, light intensity did alter the expression of many genes (Fig. 2 and Supplementary Fig. S1). Therefore, we performed differential expression analysis on the full *Chlamydomonas* transcriptome and proteome at each timepoint (Supplementary Data Set 5). Hundreds of genes were differentially expressed relative to the ML control population at both the mRNA and protein levels, even at the −2 timepoint when all three populations had been in the dark for 10 h (Fig. 3, A and B). Thus, acclimation to different intensities of diurnal light influences *Chlamydomonas*' biology in both the daytime and the nighttime.

We found that more mRNAs were differentially expressed in the LL population than in the HL population (Fig. 3A), which is at least partially attributed to the decreased synchrony of the LL population (Fig. 1, F to H). If some cells in the population are delayed in progressing through the cell cycle due to decreased growth, those cells would be delayed in the programmed expression of their genes across the diurnal cycle. At the population level, this would appear as a dampening of the peak expression signal and/or residual low-level expression outside of peak times relative to the synchronized ML control population. We observed such a dampening for mRNAs in many clusters, both for the LL population and, to a lesser extent, for the HL population (Fig. 2G).

For both the LL and HL populations, the greatest number of changes was observed at the +2 timepoint. Dawn and the onset of excess light are both known to induce massive transcriptional changes in *Chlamydomonas* (Mettler et al. 2014) and to cause significant changes in mRNA half-life (Salvador et al. 1993; Salvador and Klein 1999; Durnford et al. 2003; Smith et al. 2024). Our results show that even after prolonged diurnal photoacclimation, light intensity has the greatest impact on the transcriptome at the start of each day.

On the other hand, the number of changes to the proteome was quite similar at each timepoint (Fig. 3B). In addition, proteins from the same clusters were often increased or decreased at all five timepoints, suggesting that diurnal photoacclimation results in constitutive changes in protein abundance across the diurnal cycle. Comparative analysis revealed that a substantial proportion of the changes to the proteome were constitutive: 13% of the proteomic responses to diurnal LL and 24% of the proteomic responses to HL occurred at all five timepoints (Supplementary Fig. S4). In contrast, only 1% of mRNA changes were maintained across all five timepoints. This difference likely reflects the increased rhythmicity of the transcriptome relative to the proteome (Fig. 2C) and inherent differences in the half-lives of mRNAs and proteins.

We noted that differentially expressed mRNAs often belonged to a different set of clusters than did the differentially expressed proteins at a particular time (Fig. 3, A and B), suggesting that changes in the transcriptome often did not yield a corresponding change in the proteome. We determined the number of genes that were differentially expressed at both the mRNA and protein levels simultaneously or after a 4-h delay, as we had found that rhythmic proteins peaked later than their cognate transcripts did (Fig. 2F). Indeed, we found that most changes occurred only at the mRNA or protein level (Supplementary Fig. S5). Only ∼11% of light-responsive gene expression changes at a given time occurred at both the mRNA and protein levels, even when we considered a 4-h delay. While methodological differences undoubtedly contribute to this lack of overlap (e.g. differences in the dynamic range of RNA-Seq vs. TMT proteomics, differences in how the significance of changes in abundance was assessed for mRNAs vs. proteins), these results also speak to characteristic differences in the roles of mRNA and protein abundance in the cell and how they are controlled (de Sousa Abreu et al. 2009; Maier et al. 2009; Vogel and Marcotte 2012; Liu et al. 2016).

We sought to generate a core list of HL-responsive genes from our data. We reasoned that gene expression changes which occurred in both the HL and LL populations likely reflect differences in productivity or synchrony relative to the ML control population rather than light-specific responses. Therefore, we defined core HL-responsive mRNAs and proteins as either 1) significantly increased in HL and unchanged or decreased in LL, or 2) significantly decreased in HL and unchanged or increased in LL. These criteria filtered out hundreds of gene expression changes, especially at the mRNA level. For example, only 453 of the 735 mRNAs (61%) significantly increased at the +2 timepoint were specific to the HL-acclimated population, whereas 170 of the 190 proteins (89%) significantly increased at that time were specific to the HL-acclimated population (Supplementary Fig. S6A). This suggests that the transcriptomic analysis was more sensitive to synchrony-related population-level gene expression signals than was the proteomic analysis.

In total, 1,665 genes had HL-specific responses in transcript or protein abundance at some point over the diurnal cycle—roughly 10% of the *Chlamydomonas* genome (Fig. 3C, Supplementary Data Set 6). The gene expression changes include expected responses such as increases in the expression of light stress and light-inducible genes (e.g. *LHCSRs*, *ELIPs*) and decreases in photosystems and LHCs. In addition, CiliaCut genes—genes retained only in ciliated organisms (Merchant et al. 2007)—were significantly enriched in the core HL-responsive list (Supplementary Fig. S6B, *P* = 3.3 × 10^–15^), and many microtubule- and dynein-related genes showed an HL-specific increase in transcript abundance (Fig. 3C), suggesting that diurnal HL triggers a motility response in *Chlamydomonas* (Mayer 1968; Arrieta et al. 2017). We also observed an HL-specific increase in LCR1 protein abundance at the beginning of the day, a Myb-like transcription factor previously shown to control the expression of not only genes involved in *Chlamydomonas*' CO_2_ concentrating mechanism (CCM), but also those involved in photoprotection (Yoshioka et al. 2004; Arend et al. 2023). This was one of 24 known and predicted regulators curated by Arend et al. that exhibited HL-specific gene expression changes (Supplementary Fig. S6C). LCR1's target genes (*HLA3*, *CAH4*, *NAR1B*, and *LHCSR3*) also exhibited HL-specific increases in expression, demonstrating that this list could help to identify targets of the unstudied regulators in future work.

### Carbon fixation appears to be limited by CO_2_ availability in ***Chlamydomonas* cells acclimated to diurnal HL**

**Figure 4.**
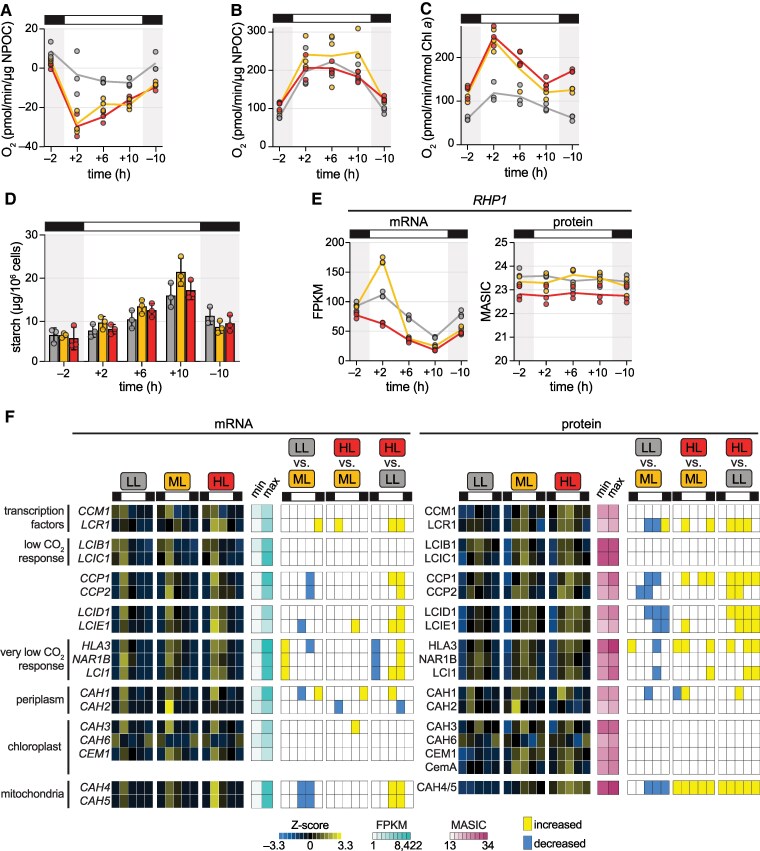
Carbon fixation appears to be limited by CO_2_ availability in *Chlamydomonas* cells acclimated to diurnal HL. Physiology and gene expression of *Chlamydomonas* populations acclimated to diurnal low light (LL, grey), moderate light (ML, yellow), and high light (HL, red). **A)** Rate of O_2_ consumption by cells collected at the designated timepoints from three replicate experiments (*n* = 3) and assayed in the dark for 5 min, normalized to NPOC as a proxy for biomass. **B)** Rate of O_2_ evolution by cells collected at the designated timepoints from three replicate experiments (*n* = 3) and assayed at 1,000 µmol photons m^−2^ s^−1^ for 3 min with excess CO_2_, normalized to NPOC as a proxy for biomass. **C)** Rate of O_2_ evolution by cells collected at the designated timepoints from three replicate experiments (*n* = 3) and assayed at 1,000 µmol photons m^−2^ s^−1^ for 3 min with excess CO_2_, normalized to mean Chl *a* content. **D)** Cellular starch content measured for three experimental replicates (*n* = 3). Error bars represent the standard deviation from the mean. **E)**  *RHP1* gene expression as a proxy for relative intracellular CO_2_ concentration. mRNA abundance (FPKM) and protein abundance (MASIC value) was determined for three experimental replicates (*n* = 3). **F)** Changes in mRNA and protein abundance of genes related to intracellular C_i_ concentration. Z-scores of mean mRNA abundances (FPKM) and Z-scores of mean protein abundances (MASIC value) across three experimental replicates (*n* = 3) are used to show patterns over time and across the three photoacclimated populations. Minimum and maximum FPKM and MASIC values are also shown to demonstrate the dynamic range. Significant increases and decreases between the three populations at a given time are indicated to the right as yellow and blue tiles, respectively. See also Supplementary Data Sets 5 and 9.

Having determined that the expression of genes related to photosynthesis, respiration, and the CCM was altered in the LL and HL populations (Figs. 2 and 3), we next monitored respiratory and photosynthetic activity. We collected cells from the photobioreactors at the diurnal timepoints of interest and assayed them in the dark to determine their capacity for respiratory O_2_ consumption and in the light in the presence of excess CO_2_ to determine their capacity for photosynthetic O_2_ evolution. We found that the rate of O_2_ consumption during dark incubation was lowest for cells collected during the night phase in all three populations, as observed previously in diurnal ML (Strenkert et al. 2019) (Fig. 4A, Tukey's HSD tests over time, *p-adj.* < 0.05). The maximum capacity for O_2_ evolution of night-time cells (assayed in the light) was also low (Fig. 4B, Tukey's HSD tests over time, *p-adj.* < 0.05). These temporal differences not only reflect how the cellular demand for ATP changes over the cell cycle (lower in G0) but also how the environment influences metabolic rate. Cooler temperatures during the night and changing CO_2_ affinity (Marcus et al. 1986) likely shape the respiratory and photosynthetic activity of the cells. Respiratory activity increased during the day but was significantly lower in LL cells (Fig. 4A, Tukey's HSD tests, *p-adj.* < 0.05). There was no significant difference in O_2_-evolution rate per biomass across the three populations during the day (Fig. 4B, one-way ANOVA tests, *P* > 0.05). However, when normalized to Chl, O_2_-evolution rate was similar for HL and ML cells and lower for LL cells (Fig. 4C, Tukey's HSD tests, *p-adj.* < 0.05). Thus, LL cells had decreased metabolic activity despite having increased Chl content (Fig. 1D) and the abundance of proteins enriched for photosynthesis and respiration GO terms (Fig. 3B).

A fraction of fixed carbon and reductant derived from photosynthesis is stored as starch, which is synthesized during the day and then degraded at night to sustain metabolism in the dark. Starch metabolism is subject to redox regulation and is under circadian control in plants and *Chlamydomonas* (Ral et al. 2006; Stitt and Zeeman 2012; Skryhan et al. 2018; Strenkert et al. 2019). Previous work has shown that starch accumulation increases upon an increase in light intensity (Mettler et al. 2014), serving as a sink for excess reductant, so we hypothesized that starch content would be highest in the HL population. However, we found that *Chlamydomonas* cells appeared to accumulate less starch over the light phase in both LL and HL (Fig. 4D, repeated measure's one-way ANOVA tests, *P* < 0.005). Cellular starch content decreased to an equivalent level in the three populations by the end of the night (−2) (repeated measure's one-way ANOVA test, *P* = 0.263). Starch degradation rate is known to be adjusted to the anticipated length of the night phase in the land plant *Arabidopsis thaliana* (Graf et al. 2010), and our results suggest that the rate of starch degradation during the night may be similarly adjusted in *Chlamydomonas* to achieve a set starch content before dawn.

Since HL cells and ML cells exhibited a similar capacity for O_2_ evolution, we hypothesized that CO_2_ concentration limits carbon fixation and starch synthesis in our HL condition. To explore this hypothesis, we investigated the expression of the CO_2_ channel RHP1, a marker for intracellular CO_2_ levels which increases at both the transcript and protein level in high CO_2_ (Soupene et al. 2002; Ruiz-Sola et al. 2023). We found that *RHP1* transcript levels were significantly decreased at the beginning of the day in the HL-acclimated population relative to the ML control, and the protein was significantly less abundant throughout the day in these cells (Fig. 4E, Supplementary Data Set 5). This suggests that intracellular CO_2_ concentrations are likely depleted under diurnal HL, as reported previously for *Chlamydomonas* cells transferred from LL to HL (Ruiz-Sola et al. 2023). Low CO_2_ concentration activates the expression of C_i_ transporters, carbonic anhydrases (CAHs), and other genes via the transcription factors CCM1 and LCR1 (Yoshioka et al. 2004; Wang et al. 2015; Mackinder 2018). We found that the abundances of LCIB1, LCIC1, and the chloroplast-localized CAH3 and CAH6 were not significantly impacted by diurnal photoacclimation (Fig. 4F). These genes are known to be induced under air levels of CO_2_ (∼0.04%), and in our growth conditions (bubbled with ambient air), increased light intensity did not boost their induction further. On the other hand, the chloroplast envelope proteins CCP1 and CCP2; the LCIB1 homologs LCID1 and LCIE1; HLA3, NAR1B, and LCI1, which are known to be induced under very low CO_2_ (<0.04%); and the mitochondria-localized CAH4 and CAH5 were significantly more abundant in the HL-acclimated population at several timepoints, including in the dark (Fig. 4F). Thus, it appears that *Chlamydomonas* cells acclimated to diurnal HL are CO_2_ limited and activate their carbon concentrating mechanisms, as reported previously (Ruiz-Sola et al. 2023), and we find that this response persists in the nighttime.

### Diurnal photoacclimation leads to differences in thylakoid membranes that persist in the dark phase

Next, we turned our attention to the morphology and composition of the thylakoid membranes, which house the photosynthetic apparatus. In *Chlamydomonas*, thylakoid membranes are organized into appressed stacks of thylakoid membranes and non-appressed, stroma-exposed membranes. The degree of stacking influences the distribution of photosynthetic proteins and the PSII repair machinery, some of which is sterically excluded from the appressed membrane regions where PSII holocomplexes are localized. Therefore, unstacking of thylakoid membranes under HL conditions is important for the degradation of photodamaged D1 subunits and the reassembly of functional PSII holocomplexes (Kirchhoff 2014; Nawrocki et al. 2021). In *Chlamydomonas*, the number of membrane layers in appressed regions is reduced in HL (Polukhina et al. 2016), and unstacking occurs within 15 min of increased irradiance (Broderson et al. 2024). However, it is unknown how thylakoid structure changes over the diurnal cycle and how acclimation to diurnal LL or HL influences stacking in *Chlamydomonas*.

**Figure 5.**
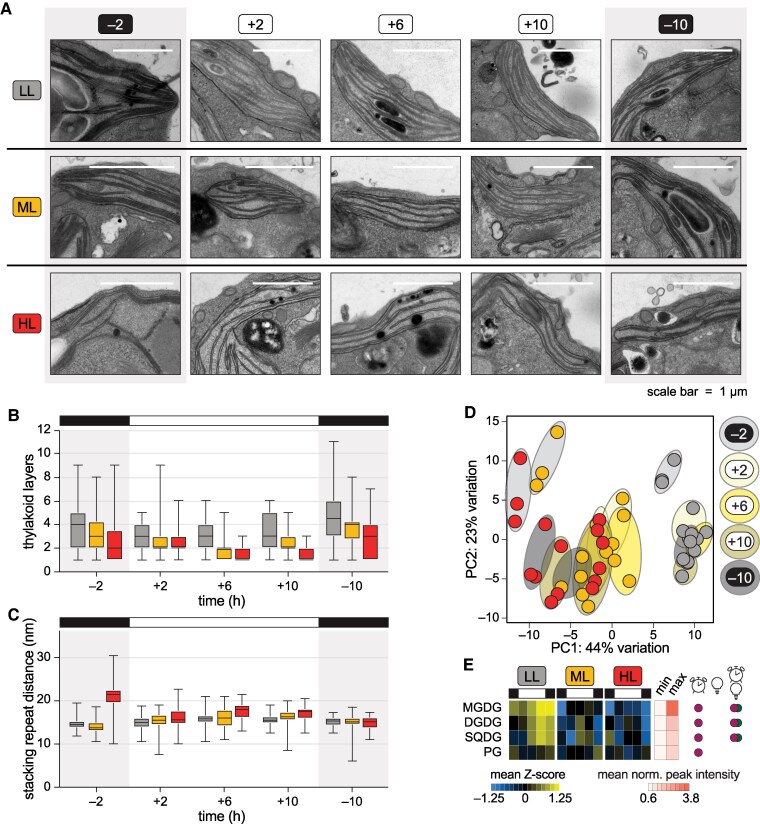
Diurnal photoacclimation leads to differences in thylakoid membranes that persist in the dark phase. Thylakoid membranes and lipidome of *Chlamydomonas* populations acclimated to diurnal low light (LL, grey), moderate light (ML, yellow), and high light (HL, red). **A)** Representative electron micrographs of thylakoid membranes from the 15 cellular states assayed in this study. Scale bars represent 1 µm. **B)** Number of thylakoid membrane layers measured for at least 37 representative thylakoid membrane regions (*n* ≥ 37) in at least 10 representative cells (*n* ≥ 10) from each of the 15 cellular states. The center line of the box plots represents the median, the box limits represent upper and lower quartiles, and the whiskers represent the minimum and maximum. **C)** Stacking repeat distance of appressed thylakoid membranes measured according to Mazur et al. 2021 for at least 37 representative thylakoid membrane regions per sample (*n* ≥ 37) in at least 10 representative cells (*n* ≥ 10) from each of the 15 cellular states. See Supplementary Fig. S7B for more details. The center line of the box plots represents the median, the box limits represent upper and lower quartiles, and the whiskers represent the minimum and maximum. **D)** PCA of lipid abundance (median-normalized peak intensities) for lipids detected by LC-ESI-MS/MS in positive ionization mode (MGDG, DGDG, SQDG, DG, DGTSA, and TG species) for three experimental replicates (*n* = 3); ellipses designate time of day. **E)** Average changes in the major classes of chloroplast lipid. Mean Z-scores of peak intensities are used to demonstrate the differences over time and across the three photoacclimated populations. Minimum and maximum peak intensities are also shown to demonstrate the dynamic range for each lipid class. Circles at right indicate a significant effect of time (clock symbol), light intensity (light bulb), or the interaction between the two by two-way mixed ANOVA (*P* < 0.05). Changes for individual lipid species within each lipid class are viewable in Supplementary Fig. S8C. See also Supplementary Figs. S7 and S8, and Supplementary Data Sets 7 and 9.

Using transmission electron microscopy (TEM), we found that thylakoid stacks appeared thin and loosely stacked in HL cells across the diurnal cycle (Fig. 5A and Supplementary Fig. S7A). Quantitative image analysis revealed that the number of thylakoid layers decreased in the daytime for all three populations but was indeed significantly lower in HL cells than in LL cells at all timepoints, even during the night (Fig. 5B, Dunn's tests, *p-adj.* < 0.05). The space between thylakoid layers, measured as the stacking repeat distance (SRD) (Supplementary Fig. S7B), was significantly higher in HL cells than in LL cells at the +6, +10, and, surprisingly, −2 timepoint, when it was often highest (Fig. 5C, Dunn's tests, *p-adj.* < 0.005). Thus, acclimation to different intensities of diurnal light resulted in changes to thylakoid structure which persist through the night, suggesting memory or anticipation of the daylight environment.

Our TEM data suggested that total thylakoid membrane area was increased in LL cells and decreased in HL cells. To test this hypothesis and determine whether thylakoid lipid composition is influenced by diurnal light intensity, we monitored the abundance of major chloroplast lipids in the photoacclimated populations using LC-ESI-MS/MS lipidomics (Supplementary Data Sets 1 and 7). Similarly to the gene expression measurements, the lipidomes of the three experimental replicates were highly correlated (pairwise *r* > 0.97, Supplementary Fig. S8A). PCA demonstrated that light intensity had a major influence on the lipidome: the first principal component (accounting for >40% of the variation) clustered the data primarily by the diurnal light intensity, rather than by time (Fig. 5D and Supplementary Fig. S8B). We found that LL cells had increased levels of thylakoid lipids over the diurnal cycle relative to HL cells, especially monogalactosyldiacylglycerol (MGDG), digalactosyldiacylglycerol (DGDG), and sulfoquinovosyldiacylglycerol (SQDG) (Fig. 5E and Supplementary Fig. S8C). As MGDG and DGDG constitute ∼80% of total thylakoid lipids (Yoshihara and Kobayashi 2022), our lipidomics data are consistent with the total thylakoid membrane area being increased under diurnal LL. In summary, we found that acclimation to different diurnal light intensities has a major impact on thylakoid architecture and lipid content, causing changes that persist across all stages of the cell cycle.

### Photosystems and their associated LHCs are co-expressed and are decreased across the diurnal cycle in HL-acclimated cells

**Figure 6.**
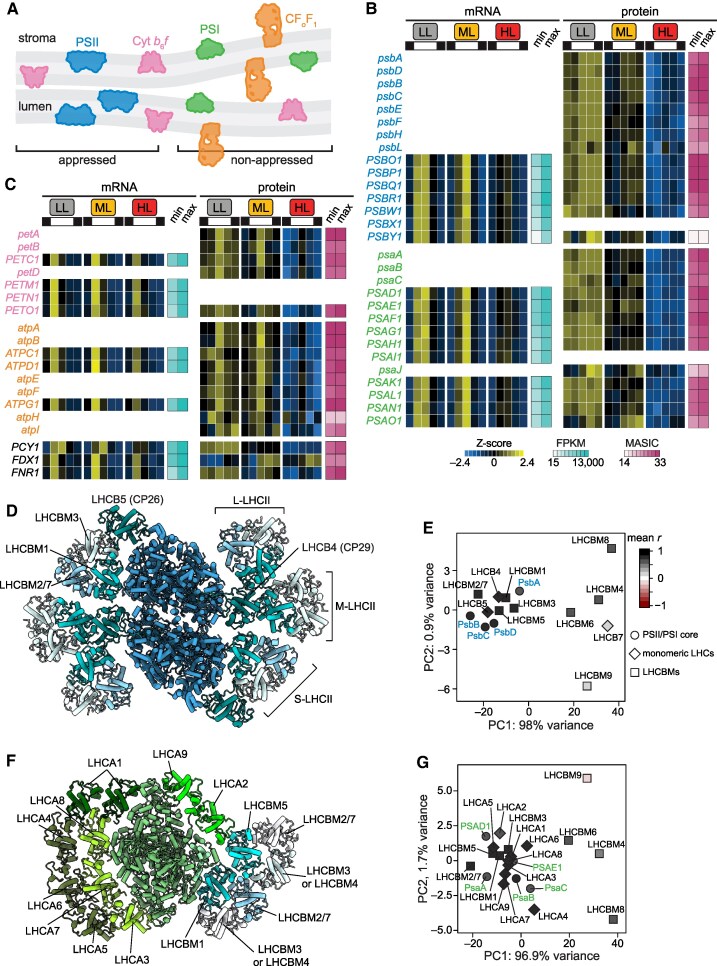
Photosystems and their associated LHCs are co-expressed and are decreased across the diurnal cycle in HL-acclimated cells. Gene expression of *Chlamydomonas* populations acclimated to diurnal low light (LL, grey), moderate light (ML, yellow), and high light (HL, red). **A)** Schematic illustrating segregation of PSII to appressed thylakoid membrane regions and of PSI and CF_o_CF_1_ to non-appressed regions. **B)** Changes in photosystem mRNAs and proteins. Gene names are colored by complex as in **(A)**. Z-scores of mean mRNA abundances (FPKM) and Z-scores of mean protein abundances (MASIC value) across three experimental replicates (*n* = 3) are used to show patterns over time and across the three photoacclimated populations. Minimum and maximum FPKM and MASIC values are also shown to demonstrate the dynamic range. **C)** Changes in mRNAs and proteins for other components of the photosynthetic electron transfer chain, represented as in **(B)**. Gene names are colored by complex as in **(A)** or are black for mobile carriers. **D)** Structure of the C_2_S_2_M_2_L_2_ PSII supercomplex isolated from *Chlamydomonas* (PDB ID 6KAD), showing the arrangement of LHCII heterotrimers and monomers around the PSII core dimer (C_2_). The composition of the S-LHCII trimer has been determined, while that of the M- and L-LHCII trimers has not been confirmed (Shen et al. 2019; Sheng et al. 2019). **E)** PCA of the abundances of LHCIIs and PSII core proteins (MASIC values) across all 45 samples (15 cellular states from three experimental replicates). Points are shaded by their mean pairwise Pearson correlation coefficients (*r*) to the four listed PSII core proteins; pairwise comparisons to self are not included in this mean. Core proteins are shown as circles, monomeric LHC proteins as diamonds, and major LHCII proteins as squares. **F)** Structure of the PSI-LHCI-LHCII supercomplex isolated from *Chlamydomonas* (PDB ID 7D0J), showing the arrangement of LHCI monomers and the association of LHCII trimers around the PSI core. **G)** PCA of the abundances of LHCBMs, LHCAs, and PSI core proteins (MASIC values) across all 45 samples (15 cellular states from three experimental replicates). Points are shaded by their mean pairwise r to the five listed PSI core proteins; pairwise comparisons to self are not included in this mean. Core proteins are shown as circles, monomeric LHC proteins as diamonds, and major LHCII proteins as squares. See also Supplementary Figs. S9 and Supplementary Data Set 5.

While PSII is localized to appressed membrane regions, PSI and the chloroplast ATP synthase (CF_o_F_1_) are segregated to non-appressed membranes and the cytochrome (Cyt) *b*_6_*f* complex is distributed throughout the thylakoid membrane system (Andersson and Anderson 1980; Wietrzynski et al. 2020) (Fig. 6A). We wondered whether the observed light intensity-dependent changes in thylakoid membrane area and stacking had an impact on the accumulation of these complexes. We found that mRNA abundances for all four complexes as well as for the mobile electron carriers plastocyanin, ferredoxin, and the ferredoxin-NADP^+^ reductase peaked in the morning and midday, suggesting coordinated biogenesis independent of the target membrane region (Fig. 6, B and C). The abundances of Cyt *b*_6_*f* and CF_o_F_1_ subunits peaked shortly after their cognate transcripts at the +6 timepoint. Cyt *b*_6_*f* is thought to be a limiting factor in photosynthetic electron transfer, and its increased abundance could contribute to the peak in O_2_ evolution capacity that we observed during the day (Fig. 4B). In contrast, the abundances of photosystem subunits were less dynamic overtime. Most PSII and PSI proteins were significantly decreased in HL-acclimated cells throughout the diurnal cycle, including during the dark phase, regardless of their distinct distribution within the thylakoid membrane system (Supplementary Fig. S9A). Plastocyanin and PETO1 were also significantly less abundant in HL-acclimated cells at all timepoints, whereas other subunits of Cyt *b*_6_*f* and CF_o_F_1_ showed only transient significant decreases upon acclimation to diurnal HL (Supplementary Fig. S9A).

The *Chlamydomonas* photosystems are surrounded by Chl-binding LHC antenna proteins, which absorb light energy and direct it to the reaction centers. The monomeric LHCII antenna proteins LHCB4 (CP29) and LHCB5 (CP26) bind directly to the PSII core monomer (C) at a 1:1 stoichiometry (Fig. 6D). In addition, heterotrimeric LHCIIs composed of the major LHCII proteins, LHCBMs, associate with various affinities to PSII. S-LHCII (strongly associated trimers) attach to LHCB5 and CP43, while the attachment of M-LHCII (moderately associated trimers) and L-LHCII (loosely bound trimers) is mediated by LHCB4 (Shen et al. 2019; Sheng et al. 2019). Structural analysis of C_2_S_2_M_2_L_2_ PSII supercomplexes isolated from *Chlamydomonas* grown mixotrophically under LL (20 to 50 µmol photons m^−2^ s^−1^) has revealed that the S-LHCII trimers are primarily composed of LHCBM1, LHCBM2/7 (which are identical proteins encoded by two independent genes), and LHCBM3 (Shen et al. 2019; Sheng et al. 2019). The composition of the M- and L-LHCII trimers remains to be determined.

We found that the pattern of expression for LHCB4, LHCB5, and LHCBM1–8 mirrored that of the PSII subunits in our experiments: mRNAs accumulated midday, and proteins were decreased in the HL-acclimated cells across the diurnal cycle (Supplementary Fig. S9B). PCA and pairwise Pearson correlations of these proteins across all samples showed that the monomeric LHCB4 and LHCB5 were co-expressed with the PSII core proteins and with one another, as were LHCBM1, LHCBM2/7, LHCBM3, and LHCBM5 (Fig. 6E and Supplementary Fig. S9C). Each of these LHCBMs has been identified in trimers associated with either PSII or PSI (Fig. 6, D and F) (Takahashi et al. 2006, 2014; Drop et al. 2014a, 2014b; Shen et al. 2019; Huang et al. 2021; Pan et al. 2021). PCA and pairwise Pearson correlations also group LHCBM1, LHCBM2/7, LHCBM3, and LHCBM5 with PSI core subunits and LHCA antenna proteins, consistent with their recruitment to PSI in State 2 (Fig. 6G and Supplementary Fig. S9D). Upon phosphorylation by STT7 kinase, LHCBM1 and LHCBM5 engage in a specific interaction with PSI and mediate the binding of LHCII trimers to the PSI core (Pan et al. 2021). The recruitment of LHCBM2/7 and LHCBM3 to the PSI-LHCI-LHCII supercomplex may be a function of their high abundance in the thylakoid membrane. The other LHCII proteins (LHCBM4, LHCBM6, LHCBM8, and LHCBM9), which are not known to be present in trimers, had lower detection on average, and PCA and pairwise Pearson correlations separate them from both PSII and PSI core proteins. Although this protein co-expression analysis is consistent with the view that LHCBM1, LHCBM2/7, LHCBM3, and LHCBM5 are the primary antenna proteins present in the LHCII trimers, detection by TMT proteomics cannot be used to determine protein stoichiometries.

### Light stress is dynamic over the daytime and may depend on cell cycle stage

**Figure 7.**
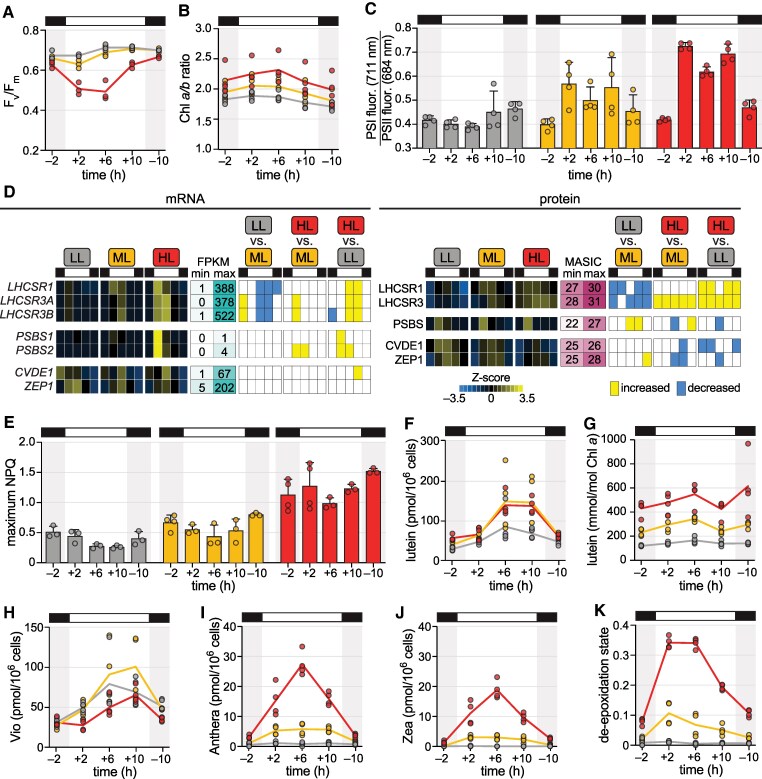
Light stress is dynamic over the daytime. Physiology and gene expression of *Chlamydomonas* populations acclimated to diurnal low light (LL, grey), moderate light (ML, yellow), and high light (HL, red). **A)** Maximum photochemical efficiency of PSII (*F_v_/F_m_*) determined for three experimental replicates (*n* = 3). **B)** Chl *a*/*b* ratio for four experimental replicates (*n* = 4). **C)** Approximate PSI fluorescence (711 nm) relative to PSII fluorescence (684 nm) measured at 77 K for cells sampled from four experimental replicates (*n* = 4). Error bars represent the standard deviation from the mean. Full Chl fluorescence spectra are viewable as Supplementary Fig. S10A. **D)** Changes in mRNA and protein abundance for qE- and qZ-related genes. Z-scores of mean mRNA abundances (FPKM) and Z-scores of mean protein abundances (MASIC value) across three experimental replicates (*n* = 3) are used to show patterns over time and across the three photoacclimated populations. Minimum and maximum FPKM and MASIC values are also shown to demonstrate the dynamic range. Significant increases and decreases between the three populations at a given time are indicated to the right as yellow and blue tiles, respectively. **E)** Maximum NPQ of cells sampled from at least three experimental replicates (*n* ≥ 3) measured from 50 to 1,500 µmol photons m^−2^ s^−1^ actinic light. See Methods section for more details. Error bars represent the standard deviation from the mean. **F)** Cellular lutein content for four experimental replicates (*n* = 4). **G)** Lutein content per Chl *a* for four experimental replicates (*n* = 4). **H)** Cellular Vio content for four experimental replicates (*n* = 4). **I)**Cellular Anthera content for four experimental replicates (*n* = 4). **J)** Cellular Zea content for four experimental replicates (*n* = 4). **K)** De-epoxidation state of xanthophylls estimated as (0.5Anthera + Zea)/(Vio + Anthera + Zea) for four experimental replicates (*n* = 4). See also Supplementary Fig. S10 and Supplementary Data Sets 5, 8, and 9.

To determine how efficiently absorbed light energy is used for photochemistry, we measured the maximum photochemical efficiency of PSII, or *F_v_/F_m_*. All three populations exhibited high *F_v_/F_m_* (∼0.7) during the night, although it was slightly (but significantly) lower for the HL population than for the LL population (Fig. 7A, Tukey's HSD test, *p-adj.* = 0.035). *F_v_/F_m_* of the HL population decreased significantly at the beginning of the day and then improved at the +10 timepoint (Tukey's HSD tests over time, *p-adj.* < 0.005). This recovery suggests that the HL cells increase the rate of PSII repair and/or alter their photoprotective strategy to reduce the amount of PSII damage in the latter half of the day.

As discussed, one way to reduce photodamage is to decrease the size of the light-harvesting antenna. We monitored changes in the ratio of Chl *a* and *b* (Chl *a*/*b*) as a proxy for antenna size, as Chl *b* is exclusively localized to LHC proteins. As compared to the LL population, the HL population exhibited a significantly higher Chl *a*/*b* ratio than that of the LL population at the +2 and +6 timepoints, suggesting reduced antenna size (Fig. 7B, Tukey's HSD tests, *p-adj.* < 0.05). While the Chl *a*/*b* ratio appeared to decrease somewhat from the +6 to the +10 timepoint, the changes over time were not significant (one-way ANOVA tests, *P* > 0.05). On the other hand, we noted that the abundance of LHCBM9 increased substantially at the +10 timepoint in the HL and ML populations, distinguishing it from the other LHC proteins (Supplementary Fig. S9). LHCBM9 is known to accumulate upon prolonged stress conditions and replace other LHCBM proteins in the antenna, thereby enhancing energy dissipation and stabilizing PSII (Grewe et al. 2014; Rochaix and Bassi 2019). We hypothesize that increased LHCBM9 at the end of the day could contribute to the recovery in PSII efficiency observed in the HL-acclimated cells.

Phosphorylation by STT7 kinase not only mediates the association of LHCII trimers with PSI during qT, it is also thought to increase electrostatic repulsion between adjacent thylakoid membranes (Chow et al. 2005; Fristedt et al. 2009; Rochaix 2014; Puthiyaveetil et al. 2017; Iwai et al. 2018; Wood et al. 2019), which could contribute to changes in thylakoid structure in HL. To assess the contribution of qT to photosynthetic efficiency and thylakoid morphology, we measured Chl fluorescence emission at 77 K (Fig. 7C and Supplementary Fig. S10A). There was almost no change in the spectrum of LL cells over time, indicating that the cells remain in State 1 (more excitation of PSII than PSI). For the ML and HL populations, the PSI fluorescence (∼711 nm) increased relative to PSII fluorescence (∼684 nm) during the day (Welch's ANOVA tests, *P* < 0.05). These changes can indicate not only an increase in the excitation energy transferred to PSI (transition to State 2) but also a decreased amount of PSII fluorescence due to photoinhibition. We also found that the relative PSI fluorescence was significantly decreased at the +6 timepoint compared to the beginning of the day for the HL population (Games-Howell test, *p-adj.* = 0.001). The emission spectra for both the ML and HL cultures returned to the LL-state (State 1) during the night, demonstrating that the observed energy redistribution is completely reversible. Importantly, thylakoid membranes remain less stacked in HL cells relative to LL cells during the night even in the absence of qT (Fig. 5). This suggests that LHCII phosphorylation is not necessary for maintaining low thylakoid stacking, as deduced previously from studies of the *Chlamydomonas stt7* kinase mutant (Broderson et al. 2024). Thylakoid stacking is also mediated by the abundance of LHCII regardless of phosphorylation state (Armond et al. 1976; Bassi et al. 1985; Simidjiev et al. 2000; Mussgnug et al. 2005; Belgio et al. 2015), so HL-acclimated cells may exhibit lower stacking than LL-acclimated cells in the night by maintaining a smaller population of LHCII proteins in their thylakoid membranes. qT during the day may contribute to the further reduction of stacking during the light phase.

Next, we determined the expression patterns of genes involved in qE. We found that while *LHCSRs* were expressed at all light intensities, mRNA and protein abundance increased with increasing light intensity as expected (Fig. 7D). LHCSR1 and LHCSR3 proteins peaked early and midday, respectively, and they remained abundant at the other timepoints examined, as observed previously (Strenkert et al. 2019; Nawrocki et al. 2020). On the other hand, PSBS only accumulated transiently, and it reached a similar maximum abundance regardless of light intensity. Although PSBS is an essential protein for activating the qE mechanism in land plants (Li et al. 2000), the function of *Chlamydomonas* PSBS orthologs is unclear. We found that NPQ capacity was highest for the population acclimated to diurnal HL (Fig. 7E, Welch's ANOVA tests, *P* < 0.05), and, in contrast to qT, it was maintained in the dark phase, as previously reported (Strenkert et al. 2019; Nawrocki et al. 2020). Since PSBS levels did not correlate with NPQ capacity, our results support the hypothesis that PSBS is not directly involved in NPQ in *Chlamydomonas*.

It has also been shown that the energy-dissipative carotenoid Zea is not essential for the qE component of NPQ in *Chlamydomonas* (Niyogi et al. 1997). Lutein, on the other hand, can form a radical cation in the LHCSR3 protein, potentially driving qE in the alga (Bonente et al. 2011; Tian et al. 2019; Zheng et al. 2024). To investigate this, we monitored these and other pigments by HPLC (Fig. 7, F to J, Supplementary Figs. S10, B to J, Supplementary Data Set 8). Lutein content scaled with cell size over the diurnal cycle and was comparable between the ML and HL populations, as was the case for Vio, α-carotene, and β-carotene (Fig. 7, F and H, Supplementary Fig. S10, B and C). However, when normalized to Chl *a*, lutein abundance was correlated with NPQ capacity: it was significantly higher in the HL population than in the LL population throughout the diurnal cycle (Fig. 7G, Tukey's HSD tests, *p-adj.* < 0.005). Antheraxanthin (Anthera) and Zea were significantly higher in the HL cells at all timepoints (Fig. 7, I and J, Games-Howell tests, *p-adj.* < 0.05), but unlike lutein, Anthera and Zea peaked midday and then declined significantly at the +10 timepoint (Games-Howell tests over time, *p-adj.* < 0.05). As a result, the de-epoxidation state of the HL cells was highest early- and midday, dropped substantially at the +10 timepoint, and then remained relatively low during the night (Fig. 7K, Games-Howell tests over time, *p-adj.* < 0.0005). This suggests that qZ contributes to NPQ mainly during the early and midday periods and that CVDE1 activity is decreased in the latter half of the day. Since CVDE1's activity is responsive to ΔpH, these results may indicate that the pH gradient has relaxed at the +10 timepoint. Furthermore, since lutein content was more closely correlated with NPQ capacity than the de-epoxidation state was, these time course data provide further evidence that lutein is primarily responsible for the qE component of NPQ in *Chlamydomonas*.

Taken together, our results demonstrate dynamic regulation of qT, qE, and qZ to orchestrate a photoprotective quenching strategy tailored not only to the intensity of daylight typically experienced by the cells but also to the time of day.

## DISCUSSION

We have presented an integrative systems analysis of the model unicellular alga *Chlamydomonas* acclimated to challenging light intensities over the diurnal cycle. By monitoring synchronous populations under conditions that simulate our natural environment (warm, bright days and dark, cool nights), we uncovered distinct, physiologically relevant responses to limiting and excess light in both the light and dark phases with high signal-to-noise.

Diurnal rhythms in gene expression and physiology are driven by several overlapping forces in *Chlamydomonas*: 1) the periodic availability of photosynthetically active radiation and heat, which restricts photosynthesis to the daytime and drives changes in metabolic rate and redox state 2) the resulting coordination of cell cycle progression with time of day that synchronizes populations (Heldt et al. 2020; Umen and Liu 2023), 3) the periodic presence of light signals, which regulate downstream effectors through transcriptional and posttranscriptional mechanisms (Johnson et al. 1991; Greiner et al. 2017), and 4) an endogenous circadian oscillator, which controls phototaxis, chemotaxis, adhesion, starch metabolism, UV sensitivity, and the expression of several nuclear and plastid genes (Mittag et al. 2005; Matsuo and Ishiura 2010). Our results suggest that the concert of these forces is sufficient to maintain robust diurnal timing of gene expression genome-wide in the face of energy limitation and light stress. 91% of transcripts accumulated with a diurnal rhythm, and their phases were largely maintained regardless of the intensity of diurnal light (Fig. 2). These robust rhythms occurred even though some diurnal signals that *Chlamydomonas* would encounter in the natural environment are missing under our controlled experimental conditions (e.g. wavelengths other than those corresponding to red and blue light). For example, phase resetting of the circadian clock is responsive not only to red and blue light in *Chlamydomonas*, but also to green and violet light (Johnson et al. 1991; Kondo et al. 1991; Niwa et al. 2013; Forbes-Stovall et al. 2014; Petersen et al. 2022). Thus, providing a broader spectrum of illumination may increase the strength and prevalence of diurnal rhythms even further. While rhythms were more difficult to detect in the proteomics data (12% to 28% of nucleus-encoded proteins and 27% to 66% of chloroplast-encoded proteins accumulated with a significant diurnal rhythm), proteins that did show significant rhythmicity also peaked at similar times regardless of light intensity. The fact that a greater proportion of chloroplast-encoded proteins accumulated with a diurnal rhythm is consistent with previous works showing that the circadian clock has greater control over chloroplast gene expression than over nuclear gene expression in *Chlamydomonas* (Breidenbach et al. 1988; Leu et al. 1990; Salvador et al. 1993, 1998; Carter et al. 2004; Kucho et al. 2005; Idoine et al. 2014). In addition, diurnal changes in redox state in the chloroplast add an additional level of genetic regulation that is largely absent from the nucleus (Salvador and Klein 1999).

Although the timing of gene expression was resilient to limiting and excess light in *Chlamydomonas*, daylight intensity did alter the expression of hundreds of genes (Fig. 3), even during the night when the three populations were subjected to identical environmental conditions (dark, 18 °C, bubbled with ambient air). Which transcripts were differentially expressed between the three photoacclimated populations depended on the time. For example, transcripts that were significantly higher in the HL population relative to the ML control at the end of the night were different from those that had been higher at the beginning of the night or at the end of the day. Thus, transcriptomic differences in the night were not simply the result of elevated mRNA pools persisting in the cell, but rather reflected increased transcription or reduced mRNA decay of new gene targets during the dark phase. Future work should interrogate how altered expression of these loci during the night phase contributes to photoacclimation, and how acclimation to limiting or excess light may influence transcription rate or mRNA decay at these loci.

In addition to diurnal changes, we found that the *F_v_/F_m_* and de-epoxidation state also change between the beginning, middle, and end of the day even when light intensity remains constant (Fig. 7). The *F_v_/F_m_* of HL-acclimated cells recovers almost completely at the +10 timepoint, concomitantly with a precipitous drop in the de-epoxidation state. These changes indicate that the HL cells partially overcome light stress once they reach S/M phase of the cell cycle. Studies on marine photosynthetic microorganisms have observed similar recoveries in the afternoon, perhaps speaking to this phenomenon as a common feature of the daily exposure to light stress (Brown et al. 1999). Future work should interrogate whether PSII repair activity is enhanced in the latter half of the day, whether LHCBM9 accumulation at this time is required for the observed recovery (Supplementary Fig. S9), or whether entry into S/M phase itself provides some relief from excess light.

Despite these dynamics in gene expression and photodamage, we found that most photoprotection strategies are constitutive in cells acclimated to diurnal HL. HL-acclimated cells maintained loosely stacked thylakoid membranes and reduced thylakoid lipid content (Fig. 5), their photosystems were less abundant (Fig. 6 and Supplementary Fig. S9), their Chl content was decreased (Figs. 1D), their LHCSR3 abundance and NPQ capacity were increased (Fig. 7, D and E), and their lutein content was higher on a per-Chl basis (Fig. 7G) across the diurnal cycle, even after 10 h of dark. Persistence of these phenotypes through the nighttime may be expected given that the associated proteins and membranes are quite stable in the absence of light stress and that the cells are in a quiescent G0 phase of limited biosynthesis during the cool darkness of night. This physiological memory of the previous day is likely advantageous for the alga to prepare for tomorrow when daylight intensity is predictable. Yet, it could come at a cost when daylight intensity varies from day to day, raising questions about how diurnal photoacclimation impacts fitness and how long it takes for the acclimatory phenotypes to manifest.

Integration of our system-wide measurements on cells acclimated to diurnal HL has contributed to our understanding of the mechanisms of photoprotection in *Chlamydomonas*. For example, our findings suggest that LHCII abundance as well as LHCII phosphorylation both contribute to changes in thylakoid membrane architecture (Figs. 5 and 7C, Supplementary Fig. S9B). In addition, our data provide further evidence that PSBS is not directly involved in NPQ in *Chlamydomonas*, and that lutein allows for the constitutive LHCSR-dependent NPQ observed (Fig. 7). The pattern of Zea accumulation suggests that qZ contributes to NPQ mainly during the early and midday periods. To further elucidate light stress responses in diurnal conditions, we include a core list of HL-responsive transcripts and proteins at each of the five timepoints examined, comprising 1,665 genes, over half of which are not associated with functional annotations (Supplementary Data Set 6, Fig. 3C, and Supplementary Fig. S6).

In addition to light responses, our transcriptomic and proteomic data also offer insights into macromolecular homeostasis in the eukaryotic cell. For example, they reveal that rhythmically expressed transcripts peak in abundance prior to their cognate proteins, reflecting the central dogma (Fig. 2F). Moreover, transient changes in transcript abundances in response to diurnal photoacclimation often generated sustained changes in protein abundances that persisted throughout the day (e.g. Figures 4F and 7D, Supplementary Fig. S9). Only ∼11% of light-responsive gene expression changes at a given time occurred at both the mRNA and protein levels (Supplementary Fig. S5). While some of these dissimilarities can be attributed to methodological differences in capturing the transcriptome and the proteome, they are also biological (de Sousa Abreu et al. 2009; Maier et al. 2009; Vogel and Marcotte 2012; Liu et al. 2016). Modest correlation between the two is routinely reported (Roch et al. 2004; Schwanhäusser et al. 2011; Lan et al. 2012; Marguerat et al. 2012; Hsieh et al. 2013; Eichelbaum and Krijgsveld 2014; Ponnala et al. 2014; Jovanovic et al. 2015) as transcripts and proteins are fundamentally different in their form, function, and regulation. Upon a stimulus, mRNA abundance typically changes by orders of magnitude to influence ribosomal occupancy, a phenomenon referred to as superinduction (Lee et al. 2011; Barry et al. 2017). Massive changes in transcript abundance are mediated not only through transcriptional activation and repression but also post-transcriptionally through changes in mRNA half-life, which has been shown to decrease up to 5-fold in response to light (Salvador et al. 1993; Salvador and Klein 1999; Durnford et al. 2003; Smith et al. 2024). The dynamic range of protein abundances is typically much smaller than that of their cognate transcripts, as protein abundance is tightly constrained to maintain proteostasis. Since cell volume roughly doubles over the diurnal cycle in our experiments, the absolute abundance of a protein would also only need to double to maintain its cellular concentration. Proteins are more often regulated by posttranslational modifications: phosphorylation (Reiland et al. 2011; Tan et al. 2020; Longoni and Goldschmidt-Clermont 2021), reduction/oxidation of cysteines (Pérez-Pérez et al. 2017; Wakao and Niyogi 2021; Zimmer et al. 2021), acetylation of lysines (Füßl et al. 2022), and protonation of side chains (Li et al. 2004; Ballottari et al. 2016; Tian et al. 2019) induce rapid, reversible changes in conformation, enzyme activity, protein-protein interactions, and localization in response to changing fluence and other stimuli. Our genome-wide data and paired physiological measurements hint at widespread posttranscriptional regulation specific to different times of day and in response to different light intensities waiting to be uncovered.

Previous genome-wide expression datasets from diurnal time-courses of *Chlamydomonas* have fed powerful co-expression analyses, gene regulatory networks, and metabolic models (Salomé and Merchant 2021; Metcalf and Boyle 2022; Arend et al. 2023; Wulf et al. 2023). They have also guided functional analysis of dozens of genes, for example in recent works on the nuclear pore complex, histones, transporters, and regulators (Mosalaganti et al. 2018; Chaux et al. 2023; Huang et al. 2023; Kafri et al. 2023; Fakhimi and Grossman 2024; Lihanova et al. 2024; Liu et al. 2024). Now, by overlaying light stress onto this synchronous cell system and providing parallel measurements of not only mRNA and proteins but also lipids, bioenergetic physiology, and plastid ultrastructure across the diurnal cycle, we provide these data as a rich resource for the photosynthesis community.

## Materials and methods

### Strains and culture conditions

*Chlamydomonas reinhardtii* strain CC-5390 [CC-4351 (*cw*15–325 *mt*+) rescued with the pCB412 cosmid carrying the *ARG7* gene] was used for all experiments. Cells were precultivated in 125 mL Erlenmeyer flasks containing 50 mL Tris-acetate-phosphate (TAP) medium with a modified trace element solution (Kropat et al. 2011) in an Innova incubator (New Brunswick Scientific, NJ, USA) with constant agitation at 140 rpm, 24 °C, and continuous white light (50 to 60 μmol photons m^−2^ s^−1^). Cultures were synchronized and grown in presterilized turbidostat flat-panel FMT 150 Photobioreactors (Photon Systems Instruments, Drásov, Czechia) containing high-salt (HS) medium supplemented with a modified trace element solution (Sueoka 1960; Kropat et al. 2011) as described previously (Strenkert et al. 2019). Briefly, photobioreactors were inoculated with cells precultivated in TAP to a starting optical density (OD_680_) of ∼0.05. Cultures were aerated with filter-sterilized ambient air provided by an aquarium pump, which also mixed cultures with airlift. Cells were grown under 12-h-light/12-h-dark cycles, where temperature was set to 28 °C during the light phase and 18 °C during the dark phase, and illumination was provided by a panel of red and blue LEDs (80% blue, 20% red, LED Light Source SL 3500; Photon Systems Instruments, Drásov, Czechia). Light intensity was set to 50 μmol photons m^−2^ s^−1^ for LL, 200 μmol photons m^−2^ s^−1^ for ML, and 1,000 μmol photons m^−2^ s^−1^ for HL. Cells were acclimated to the respective diurnal light condition by growing in turbidostat mode for a minimum of 2 weeks before performing the experiments. As the LL condition limited growth, cells were first grown for 1 week at ML, and then the light was changed to LL for at least 1 week before performing the experiments. At the start of each experiment, OD_680_ was set to 0.4 at the beginning of the dark phase, and the flow of media was stopped in order to monitor changes in culture density. Experimental replicates refer to independent experiments performed with independent cultures in different weeks.

### Live-cell imaging by Airyscan microscopy

10 mL of culture was collected by centrifugation at 1800 × *g* for 1 min, and cells were resuspended in 0.7% low-melting-point agarose in HS medium and mounted between slide and coverslip. Cells were observed using a Zeiss LSM 880 or a Zeiss LSM 980 microscope equipped with the Airyscan detector with a Zeiss Plan-Apochromat 63×/1.4 NA DIC M27 Oil objective (Zeiss, Oberkochen, Germany). Chlorophyll (Chl) was excited with a 633 nm laser, and fluorescence was acquired through a 645 nm longpass filter. At least 12 representative cells were imaged per condition, and one representative image was chosen for display. Image acquisition and analysis were performed using ZEN software (Zeiss, Oberkochen, Germany) and FIJI image analysis software (Schindelin et al. 2012; Schneider et al. 2012).

### Cell density, size, and estimation of culture synchrony

Cell density was continuously monitored by the bioreactors as OD_680_, and the mean OD_680_ and standard deviation across 9 experimental replicates (*n* = 9) is displayed. Cell number was determined for all experimental replicates with a Z2 Coulter Particle Count and Size Analyzer (Beckman Coulter, CA, USA) set to count particles between 4 and 14 µm in diameter. Cell diameter was determined for three experimental replicates (*n* = 3). Images of cultures immobilized with iodine were taken <15 min after each timepoint at 100× total magnification using ZEN software and diameters were estimated using FIJI image analysis software. In FIJI, images were made binary, holes were filled, and the “watershed” command was used to distinguish actively dividing daughter cells and count them as individual cells. The conversion factor of 0.44 µm/pixel (corresponding to the magnification of the image) was used to determine the diameter of particles ranging from 15 to 3,000 µm^2^ in area with a circularity factor from 0.5 to 1. The diameter of 100 cells from each of the three experimental replicates (300 cells total) is plotted for each condition.

As an additional qualitative metric of population synchrony, the fraction of cells in the population undergoing S/M phase of the cell cycle was estimated by microscopy for three experimental replicates (*n* = 3). Cells exhibiting a cleavage furrow or that had divided into two daughter cells but not yet separated from each other (not yet hatched) were considered to be dividing. At least 40 total cells were counted for each of the three experimental replicates.

### Pigment content

Pigment content was measured by HPLC. 10 mL of culture from each of 4 experimental replicates (*n* = 4) was collected by centrifugation at 2,400 × *g* for 3 min at 4 °C, and the pellets were flash-frozen in liquid N_2_ and stored at −80 °C until further processing. After thawing, 100 µL of 100% acetone was used to extract pigments from each sample by vortex for 10 min. The sample was centrifuged at 21,000 × *g* for 5 min at 4 °C, and the supernatant was transferred to a collection tube. Another 100 µL of 100% acetone was added to the pellet to extract pigment completely by vortexing for 10 min. The pellet was removed by centrifugation at 21,000 × *g* for 5 min at 4 °C, and the supernatant was added to the same collection tube as before. The extracted pigments were passed through a 0.22 µm nylon filter and transferred to an HPLC sample vial. HPLC was performed using a Spherisorb ODS1 C_18_ column (4.6× 250 mm, Waters Corporation, MA, USA) and pigment analysis was performed as previously described (Müller-Moulé et al. 2002). Pigment content was normalized to either cell number or Chl *a*. The cellular content of measured pigments (pmol/10^6^ cells) is available in Supplementary Data Set 8.

### Total organic carbon measurement

Total nonpurgeable organic carbon (NPOC) of cells was determined for three experimental replicates (*n* = 3) using a TOC-L Shimadzu Total Organic Carbon Analyzer (Shimadzu, Kyoto, Japan). 13 mL of culture was collected by centrifugation at 3,200 × *g* and 4 °C for 2 min, and the cell pellets were washed once in 10 mm Na-phosphate buffer (pH 7.0). Cell pellets were stored at −20 °C until analysis (<1 month). To digest cells, pellets were resuspended in 3 m HCl to achieve roughly 3.33× 10^7^ cells/mL acid and then incubated at 65 °C for 48 h with constant agitation. Cell digests were diluted 111-fold with MilliQ H_2_O for a final HCl concentration of 27 mm. All samples were sparged to remove inorganic carbon; some organic carbon may be purged from the sample by this method, so we report “nonpurgeable” organic carbon.

### RNA extraction

15 mL of culture from each of three experimental replicates (*n* = 3) was collected by centrifugation at 2,500 × *g* and 4 °C for 5 min. Cell pellets were resuspended in 1 mL lysis buffer containing 50 mm Tris-HCl pH 7.5, 150 mm NaCl, 15 mm EDTA pH 8, 2% (w/v) SDS, and 40 µg/mL Proteinase K (from *Tritirachium album*, Fisher Scientific, NH, USA) and mixed by gentle pipetting and inversion for 30 s. Cellular starch was removed by centrifugation at 600 × *g* for 3 min, and the lysate was transferred to a fresh 15 mL tube and flash-frozen in liquid N_2_ before storing at −80 °C. RNA from 4 to 5 frozen samples was extracted in parallel; samples were kept on ice and solutions were pre-cooled when possible. 10 mL TRIzol (Invitrogen, Thermo Fisher Scientific, MA, USA) was added to each frozen pellet and samples were gently inverted until thawed. Then, nucleic acids were extracted by adding 2 mL chloroform/isoamylalcohol (24:1, v/v) and shaking vigorously for 30 s. The sample was quickly transferred to MaXtract High Density tubes (QIAGEN, MD, USA), phases were separated by centrifugation at 1500 × *g* and 20 °C for 5 min, and the aqueous phase was carefully transferred to a fresh tube and mixed with ∼10 mL of cold ethanol (100%). The mixture was transferred onto an RNeasy Mini column (QIAGEN, MD, USA) using a vacuum manifold, washed according to manufacturer's instructions but with buffer RWT instead of RW1, DNase treated on the column, washed with buffer RWT and RPE, and eluted into 90 µL RNase-free water. In an additional step, the RNA was further purified by precipitation with 0.3 m sodium acetate (pH 7.0) and 2.5-volumes of ethanol (100%) for 30 min at −20 °C, followed by centrifugation at 16,200 × *g* and 4 °C for 30 min. The resulting pellets were washed with 70% (v/v) ethanol and resuspended in 60 µL RNAse-free water. The concentration of total RNA was estimated using a NanoDrop One (Thermo Fisher Scientific, MA, USA) to be >100 ng/µL. RNA quality was assessed by the ratios of A_260_/A_280_ ∼2.05 to 2.20 and A_260_/A_230_ > 2.00, and by using the Agilent RNA 6000 Pico Kit with a 2100 Bioanalyzer according to the manufacturer's instructions (Agilent, CA, USA).

### Transcriptomics sequencing and quantitation

Total RNA was subjected to poly(A) selection, RNA-Seq library construction, and sequencing on the Illumina HiSeq 3000 platform by the University of California Los Angeles Technology Center for Genomics and Bioinformatics (UCLA, CA, USA) using standard kits and protocols (Illumina Inc. CA, USA). Sequencing comprised 3 lanes of 50 nt reads, which generated 933 M reads total from 45 libraries (three light intensities × five timepoints × three experimental replicates). The resulting reads were mapped to the *C. reinhardtii* reference genome assembly and annotations v6.1 (https://phytozome-next.jgi.doe.gov/info/CreinhardtiiCC_4532_v6_1) (Craig et al. 2022) with STAR (v2.4.0j) using --runThreadN 4 --alignIntronMax 5000. The resulting sam formatted files were compressed, sorted and indexed with samtools (v1.16.1) using view -b -h, sort, and index, respectively. FPKMs were calculated with cuffdiff (v2.0.2) using – max-bundle-frags 1000000000 – library-type fr-firststrand. FPKM values for all 16,735 nucleus-encoded mRNAs detected are available in Supplementary Data Set 2, along with gene symbols and functional descriptions. More information about each gene can be found on https://phytozome-next.jgi.doe.gov/info/CreinhardtiiCC_4532_v6_1. Reproducibility across the three experimental replicates was assessed by calculating the pairwise Pearson correlation coefficient (*r*) for the FPKMs in all mRNAs that were detected in both replicates being compared: the cor function from the R package *stats* (v4.4.1) was applied with method = “pearson” and missing values were excluded with use = “complete.obs”.

### Protein and lipid extraction

35 mL of culture from each of three experimental replicates (*n* = 3) was collected by centrifugation at 2,400 × *g* at 4 °C for 3 min, resuspended in 200 µL Na-phosphate buffer (pH 7.0), transferred to 1.5 mL tubes, flash-frozen in liquid N_2_, and stored at −80 °C. Cell pellets were extracted using the Metabolite, Protein, and Lipid extraction (MPLex) method (Nakayasu et al. 2016; Nicora et al. 2020). The cell pellets were resuspended in Type 1 water and cold (−20 °C) chloroform/methanol (2:1, v/v) in chloroform-compatible 2 mL Sorenson MµlTI SafeSeal microcentrifuge tubes (Sorenson BioScience, UT, USA). The resulting mixture had a final ratio of 8 parts chloroform, 4 parts methanol, 3 parts water/sample. Samples were vortexed, sonicated on ice in a bath sonicator, and then incubated in an ice block with shaking for 45 min. Samples were then sonicated with a 3 mm probe (20 kHz fixed ultrasonic frequency, sonicator model FB505, Thermo Fisher Scientific, MA, USA) at 20% of the maximum amplitude for 30 s on ice within a fume hood. The polar and nonpolar phases, as well as the protein interlayer, were separated by centrifugation at 12,000 × *g* for 10 min.

The upper polar phase was removed. The protein interlayer was washed with cold 100% methanol, resuspended by vortexing, and the protein was collected by centrifugation at 12,000 × *g* for 5 min. Protein pellets were lightly dried under an N_2_ stream and stored at −80 °C until analysis. The lower nonpolar phase was collected for lipidomics analysis: the solvent was removed by drying in a speed vac, 500 µL of cold chloroform/methanol (2:1, v/v) was added, and the samples were stored at −20 °C until analysis.

### Protein digestion

8 m urea was added to each protein pellet, and a bicinchoninic acid (BCA) assay was used to determine the protein concentration (Thermo Fisher Scientific, MA, USA). Then, 10 mm dithiothreitol (DTT) was incorporated into the samples by incubating at 60 °C for 30 min with continuous agitation at 800 rpm. The samples were then diluted 8-fold before digestion in a solution containing 100 mm triethylammonium bicarbonate (TEAB), 1 mm CaCl_2_, and sequencing-grade modified porcine trypsin (Promega, WI, USA) at a trypsin-to-protein ratio of 1:50 (w/w) for 3 h at 37 °C.

Following digestion, the samples were desalted using a 4-probe positive pressure Gilson GX-274 ASPEC system (Gilson Inc., WI, USA) with Discovery C18 100 mg/1 mL solid phase extraction columns (Supelco, MO, USA). Initial conditioning was performed with 3 mL of methanol followed by 2 mL of 0.1% trifluoroacetic acid (TFA) in H_2_O, the samples were loaded onto each column, washed with 4 mL of a solution of 95:5 H_2_O:acetonitrile and 0.1% TFA, and eluted with 1 mL of 80:20 acetonitrile:H_2_O containing 0.1% TFA. The eluted samples were concentrated to approximately 100 µL using a speed vac, and another BCA assay was performed to determine the peptide concentration. 2 µg from each sample was combined to form a global pool, while 30 µg from each sample was aliquoted into new tubes for isobaric multiplexing. These samples were completely dried in a speed vac.

### TMT peptide labeling

Samples were pooled randomly into three groups called “plexes.” Each plex also contained a global pool for normalization across sets. Dried samples were diluted in 40 µL of 500 mm HEPES (pH 8.5) and labeled using an amine-reactive TMT Isobaric Mass Tagging Kit (Thermo Fisher Scientific, MA, USA) according to the manufacturer's instructions. 250 μL of anhydrous acetonitrile was added to each 5 mg reagent, which was dissolved over 5 min with occasional vortexing. Reagents (10 µL) were then added to each sample and incubated for 1 h at ambient temperature with shaking at 400 rpm. Each sample was then diluted with 30 µl 20% acetonitrile (v/v). A portion from each sample was collected as a premix, which was run on a mass spectrometer to confirm complete labeling. The samples were frozen at −80 °C until further analysis. Frozen samples were thawed, and the reaction was quenched by adding 8 μL of 5% hydroxylamine (v/v) and incubating for 15 min at ambient temperature with shaking at 400 rpm. The samples within each set were then combined and completely dried in a speed vac. Samples were then cleaned using Discovery C18 50 mg/1 mL solid phase extraction tubes as described above and once again assayed by BCA to determine the final peptide concentration. Samples were diluted in 2% ACN, 0.1% formic acid to a peptide concentration of 0.25 μg/μL for microfractionation.

Peptide mixtures were separated by high resolution reversed phase UPLC using a nanoACQUITY UPLC system (Waters Corporation, MA, USA) equipped with an autosampler. Capillary columns (200 µm i.d. × 65 cm long) were packed with 300 Å 3.0 µm p.s. Jupiter C18 bonded particles (Phenomenex, CA, USA). Separations were performed at a flow rate of 2.2 µL/min on binary pump systems using 10 mm ammonium formate (pH 7.5) as mobile phase A and 100% acetonitrile as mobile phase B. 48 µL of TMT-labeled peptide mixtures (0.25 µg/µL) were loaded onto the column and separated using a binary gradient of 1% B for 35 min, 1% to 10% B in 2 min, 10% to 15% B in 5 min, and 15% to 25% B in 35 min, 25% to 32% B in 25 min, 32% to 40% B in 13 min, 40% to 90% B in 43 min, held at 90% B for 2 min (column washed and equilibrated from 90% to 50% B in 2 min, 50% to 95% B in 2 min, held at 95% B for 2 min and 95% to 1% in 4 min). The capillary column eluent was automatically deposited every minute into 12× 32 mm polypropylene vials (Waters Corporation, MA, USA) starting at 60 min and ending at 170 min over the course of the 180 min LC run. Fractions were concatenated by collecting fractions from vial 1 to vial 24 and then returning to vial 1 back to vial 24 (etc.). Prior to peptide fraction collection, 100 µL of water was added to each vial to aid the addition of small droplets into the vial. Each vial was completely dried in a vacuum concentrator (Labconco, MO, USA) and reconstituted in 20 µL 2% acetonitrile, 0.1% formic acid and stored at −20 °C until LC-MS/MS analysis.

### TMT-labeled peptide mass spectrometry

A Thermo Dionex Ultimate 3000 UPLC system (Thermo Fisher Scientific, MA, USA) was configured to load a 5 µL injection directly onto the column at a flow rate of 200 nL/min, allowing 40 min to load the sample onto the column before the elution gradient was started. The analytical column was made using an integrated emitter capillary (75 µm i.d. × 25 cm long), packed in-house using BEH C18 media (Waters Corporation, MA, USA) in 1.7 µm particle size. Columns were heated to 45 °C using the MonoSLEEVE controller and a 15 cm heater (Analytical Sales and Services Inc., NJ, USA). Mobile phases consisted of (A) 0.1% formic acid in H_2_O and (B) 0.1% formic acid in acetonitrile with the following gradient profile (min, %B): 0, 1; 40, 1; 50, 8; 145, 25; 155, 35; 160, 75; 165, 5; 170, 95; 175, 1.

MS analysis was performed using a Q-Exactive HF-X mass spectrometer (Thermo Fisher Scientific, MA, USA) outfitted with a Nanospray Flex Ion Source (Thermo Fisher Scientific, MA, USA) ionization interface. The ion transfer tube temperature and spray voltage were 300 °C and 2.2 kV, respectively. Data were collected for 120 min following a 60 min delay from sample injection. FT-MS spectra were acquired from 300 to 1,800 m/z at a resolution of 60 K (automatic gain control (AGC) target 3× 10^6^) while the top 12 FT-HCD-MS/MS spectra were acquired in data-dependent mode with an isolation window of 0.7 m/z and a resolution of 45 K (AGC target 1 × 10^5^) using a normalized collision energy of 30% and an exclusion time of 45 s.

### TMT proteomics data analysis

LC-MS/MS datafiles were converted to mzML format using MSConvert, part of the ProteoWizard (Chambers et al. 2012) suite of software tools. MZRefinery (Gibbons et al. 2015) was employed to re-calibrate the MS level search mass values. Adjusted spectra were then searched using MS-GF+ (Kim and Pevzner 2014) with partial tryptic cleavage rules, +/− 20 ppm parent mass tolerance, static TMT 16-plex modification (+229.1629 Da) of peptide N-termini and lysine residues, dynamic oxidation (+15.9949 Da) of methionine residues, and instrument set to Q-Exactive derived data. A target/decoy approach was employed (Elias and Gygi 2007) against 32,670 protein sequences from the *Chlamydomonas reinhardtii* v6.1 genome annotation (https://phytozome-next.jgi.doe.gov/info/CreinhardtiiCC_4532_v6_1) (Craig et al. 2022) coupled with 16 commonly observed contaminants (trypsin, keratin, etc.). Peptide-to-spectrum matching (PSM) identifications were filtered to 1% FDR (10,277 decoy from 1,027,720 filter passing PSMs), matched back to the search FASTA using the Protein Coverage Summarizer tool (Protein Coverage Summarizer · bio.tools), and peptide uniqueness was assessed. Parsimony was applied such that only non-unique peptides mapping to more than one uniquely identified Gene IDs (2.1% of the overall data) were excluded from quantitation. The MASIC software tool (Monroe et al. 2008) was used to extract TMT reporter ion signal abundances within a tolerance of +/− 0.003 Da, as well as an isotopic interference assessment for precursor signals. Signals with more than 20% non-parent isotopic interference (≤0.8) were excluded from quantitation. Resulting peptides and their associated abundances (MASIC values) were log_2_-transformed, visualized, and mean central tendency normalized using InfernoRDN (Polpitiya et al. 2008). Normalized peptide abundances were de-logged, grouped by parsimony-filtered Gene IDs, and abundances summed (simple protein rollup). Resulting Gene ID abundances were log_2_-transformed and mean central tendency normalized to remove small, experimentally induced variations, resulting in the final MASIC values reported.

While 11,462 proteins were detected across the dataset, only proteins which were detected in at least two of the three experimental replicates of all 15 cellular states were analyzed (10,075 proteins total); MASIC values for these proteins are available in Supplementary Data Set 2, along with gene symbols and functional descriptions. More information about each gene can be found on https://phytozome-next.jgi.doe.gov/info/CreinhardtiiCC_4532_v6_1. Reproducibility across the three experimental replicates was assessed by calculating the pairwise Pearson correlation coefficient (*r*) for the MASIC values in all proteins that were detected in both replicates being compared: the cor function from the R package *stats* (v4.4.1) was applied with method = “pearson” and missing values were excluded with use = “complete.obs”.

### Enzymatic starch measurement

20 mL of culture from each of three experimental replicates (*n* = 3) was collected in Protein LoBind tubes (Eppendorf, Hamburg, Germany) by centrifugation at 1,650 × *g* for 10 min. Cell pellets were flash-frozen in liquid N_2_ and then stored at −80 °C until processing (<2 weeks). Samples were processed and assayed in batches by experimental replicate. Starch was extracted from the cell pellets by ethanolic extraction in three subsequent cycles. Each pellet was resuspended in 250 µL 80% (v/v) ethanol, and the suspensions were transferred to screw-cap tubes with rubber gaskets to minimize evaporation loss. Each tube was incubated at 95 °C for 30 min with constant agitation, allowed to cool to ambient temperature (∼5 min), and the pellet collected by centrifugation at 5,000 × *g* for 10 min. The supernatant was carefully removed, and the pellet was subsequently treated in the same way with 150 µL of 80% (v/v) ethanol and then with 250 µL of 50% (v/v) ethanol, respectively. The resulting pellets were hydrolyzed by resuspending in 400 µL of 0.1 m NaOH, shaking vigorously, incubating at 95 °C for 30 min with constant agitation, allowed to cool to ambient temperature, and the pellet collected by centrifugation at 5,000 × *g* for 10 min. The supernatant was carefully removed, and the pellet was resuspended in 80 µL of freshly prepared neutralization solution (0.1 m sodium acetate, with pH adjusted to 4.9 with NaOH and 0.5 m HCl) and 320 µL of MilliQ H_2_O was added to prepare the final sample volume. The starch content in each sample was determined using a Starch Assay Kit (Sigma-Adrich, MO, USA) according to the manufacturer’s instructions, but with the sample preparation replaced by the ethanolic extraction and hydrolysis described above, and the assay adjusted to smaller reaction volumes. The starch assay volume was reduced to 350 µL while maintaining reagent ratio and the glucose assay was performed in 96-well plates using 20 µL of the starch assay reaction in a total reaction volume of 200 µL. Cellular glucose levels were determined for each sample in a parallel reaction lacking amyloglucosidase, and the resulting background glucose concentration was subtracted from the final glucose concentration (cellular glucose + glucose derived from starch) to calculate starch content. Starch concentration was normalized to cell number.

### O_2_ consumption and evolution measurements

O_2_ consumption and evolution rates were measured for three experimental replicates (*n* = 3) on a standard Clark-type electrode (Hansatech Oxygraph with a DW-2/2 electrode chamber) and analyzed with Hansatech O2view software (v2.10, Hansatech Instruments Ltd, Norfolk, UK). 2 mL of cells sampled at the designated timepoints were assayed in the presence of 5 mm NaHCO_3_ under constant agitation. Respiration rates were measured as O_2_ consumption over a period of 5 min in the dark. Then, O_2_ evolution was measured during illumination with 50 μmol photons m^−2^ s^−1^ for 5 min, 200 μmol photons m^−2^ s^−1^ for 3 min, and 1,000 μmol photons m^−2^ s^−1^ for 3 min. As O_2_ evolution rates were highest during illumination with 1,000 μmol photons m^−2^ s^−1^, only those rates were reported. The rate of photosynthetic O_2_ evolution was calculated as the difference between O_2_ evolution and O_2_ consumption in the dark for each sample. O_2_ consumption and evolution rates were normalized to NPOC as a proxy for biomass or to Chl *a*.

### Transmission electron microscopy

50 mL of culture was collected by centrifugation at 1800 × *g* for 1 min, cells were resuspended in 2% glutaraldehyde in HS medium, and fixed by incubation at 4 °C with rotatory agitation in the dark for >10 h. The fixed cells were washed and post-fixed with Na-cacodylate buffer containing 1% (w/v) OsO_4_ and 1.6% (w/v) potassium ferricyanide for 1 h. The fixed cells were washed twice with cacodylate buffer for 10 min. The washed cells were then dehydrated with increasing concentrations of acetone (35% to 100%). Dehydrated cells were infiltrated and embedded in EPON resin. Sections were cut to approximately 70 to 100 nm in thickness. The thin sections were collected onto copper formvar slot grids. Sections were post-stained with 2% uranyl acetate for 7 min, followed by lead citrate for 7 min. The sections were dried and examined using a JEOL 1200 EX 100 kV TEM (JEOL, MA, USA) with an Orius CCD camera (Gatan Inc., CA, USA) or a Technai 12 120 kV FEI TEM with a Rio16 CMOS Camera (Gatan Inc, CA, USA). At least 15 representative cells were imaged from each sample, and one representative cell was chosen for display.

### Image analysis of thylakoid membranes

The characteristics of thylakoid membranes [number of thylakoid membrane layers, membrane height, and SRD were manually measured for at least 37 representative thylakoid membrane regions per sample (*n* ≥ 37) (from at least 10 representative cells per sample) using FIJI image analysis software as previously described (Mazur et al. 2021). The analysis method was modified as follows because *Chlamydomonas* thylakoid membrane architecture has less distinct grana structures than do land plants. Instead of only collecting data on thylakoid membrane stacks with three or more membrane layers, we collected data on all levels of stacking, including single, unstacked thylakoid membranes. Membrane height is defined as the distance between the top and bottom layers of the outer thylakoid membranes of a stack. SRD represents the average thickness of each thylakoid membrane and is calculated by dividing the height of the membrane stack by the number of membrane layers in the stack (Supplementary Fig. S7B). SRD was only determined for stacked thylakoid membranes (2 or more thylakoid membrane layers).

### Lipidomics analysis

Untargeted lipid detection, identification, and quantitation were achieved by LC-ESI-MS/MS analysis using a Vanquish Flex UHPLC system (Thermo Fisher Scientific, MA, USA) interfaced with a Velos-ETD Orbitrap mass spectrometer (Thermo Fisher Scientific, MA, USA). Dried lipids extracted by MPLex (described above) were reconstituted in 90% methanol and 10% chloroform (v/v) and injected onto an ACQUITY UPLC CSH column (3.0 mm × 150 mm × 1.7 µm particle size, Waters Corporation, MA, USA). Lipids were separated over a 34 min gradient elution (mobile phase A: acetonitrile:H_2_O (40:60, v/v) containing 10 mm ammonium acetate; mobile phase B: acetonitrile:isopropyl alcohol (10:90, v/v) containing 10 mm ammonium acetate) at a flow rate of 250 µL/min. The full gradient profile was as follows (min, %B): 0, 40; 2, 50; 3, 60; 12, 70; 15, 75; 17, 78; 19, 85; 22, 92; 25, 99; 34, 99; 34.5, 40. The mass spectrometer inlet and HESI source were maintained at 350 °C with a spray voltage of 3.5 kV and sheath, auxiliary, and sweep gas flows of 45, 30, and 2, respectively. Samples were analyzed in both positive and negative ionization using higher-energy collision dissociation (HCD) and collision-induced dissociation (CID) to obtain high coverage of the lipidome. Data were collected using a precursor scan of 200 to 2,000 m/z at a mass resolution of 60 k, followed by data-dependent MS/MS of the top 4 ions. Normalized collision energies for CID and HCD were 35 and 30, respectively. CID spectra were acquired in the ion trap using an activation q-value of 0.18, while HCD spectra were acquired in the orbitrap at a mass resolution of 7.5 k. LC-MS/MS raw data files were imported into the in-house developed software LIQUID (Lipid Informed Quantitation and Identification) (Kyle et al. 2017) for semi-automated identification of lipid molecular species. Confident lipid identifications were determined by examining the tandem mass spectra for diagnostic ion fragments along with associated chain fragment information. In addition, the isotopic profile, extracted ion chromatogram (XIC), and mass error of measured precursor ions were examined for lipid species. The identified lipid name, observed m/z, and the retention time from each analysis were used as the target database for feature identification across all LC-MS/MS runs to align and gap-fill the mass spectrometry data. This was achieved by aligning all datasets (grouped by ionization type) and matching unidentified features to their identified counterparts using MZmine 2 (Pluskal et al. 2010). Aligned features were manually verified, and peak intensity values were exported for statistical analysis (Kyle et al. 2016).

In total, 268 lipid species were detected: 199 in positive mode (DG, DGDG, DGTSA, MGDG, SQDG, and TG species) and 69 in negative mode (PA, PE, PG, and PI species). Peak intensity was log_2_-transformed and then median-normalized to correct for differences in material loading across samples. Median-normalized peak intensity for all lipid species detected are available as Supplementary Data Set 7. Reproducibility across the three experimental replicates was assessed by calculating the pairwise Pearson correlation coefficient (*r*) for the median-normalized peak intensities in all lipid species that were detected in both replicates being compared: the cor function from the R package *stats* (v4.4.1) was applied with method = “pearson” and missing values were excluded with use = “complete.obs”.

### *F_v_/F_m_* measurement

*F_v_/F_m_* was determined for each of three experimental replicates (*n* = 3). Roughly 10 mL of culture was collected into 25 mL Erlenmeyer flasks and dark-adapted for 15 min. Then, 350 µL of culture was aliquoted into each of 18 replicate wells of a 96-well plate per sample to measure maximum quantum efficiency of PSII using an IMAG-MAX/L MAXI Imaging PAM system equipped with ImagingWinGigE software (Heinz Walz GmbH, Effeltrich, Germany). *F_v_/F_m_* was calculated as *F_v_/F_m_* = (*F_m_* − *F*_0_)/*F_m_*, where *F_m_* is the maximum fluorescence measured immediately after a saturating pulse and *F*_0_ is the initial fluorescence of dark-adapted cells. The saturating pulse was administered at an intensity of 10 units and a pulse width of 0.48 s. Gain and damping of the instrument were set to 18 and 4, respectively, such that the F_t_ of a dark-adapted control culture was between 0.15 and 0.20. Control cultures were grown in TAP medium under 50 to 60 µmol photons m^−2^ s^−1^ (mixture of warm and cool white light) and 24 °C with constant agitation at 140 rpm in an Innova incubator, and were then kept at a lower light intensity for the duration of each experiment time course (∼20 µmol photons m^−2^ s^−1^).

### 77 K fluorescence emission spectra

Steady-state Chl fluorescence emission spectra at 77 K were measured for four experimental replicates (*n* = 4) with a FluoroMax-4 spectrophotometer (Horiba Scientific, Kyoto, Japan). 1.5 mL of culture was sampled into glass tubes and immediately frozen by submerging in liquid N_2_. The excitation wavelength was 420 nm with a 2 nm slit size. Fluorescence emission was measured from 650 to 780 nm with a 4 nm slit size. Fluorescence emission at each wavelength was measured for 0.1 s, the spectra were recorded three consecutive times for each sample, and the averaged spectra for each replicate sample were obtained. Fluorescence at each wavelength was normalized relative to the maximum fluorescence (resulting from PSII, ∼684 nm). The mean spectra and standard deviation across the 4 experimental replicates are displayed. The PSI fluorescence relative to the PSII fluorescence is reported as the ratio between the fluorescence at the 711 nm peak and the fluorescence at the 684 nm peak.

### NPQ capacity

NPQ capacity was determined by monitoring Chl fluorescence over light curves for at least three experimental replicates (*n* = 3). Around 20 mL of culture was collected into 50-mL Erlenmeyer flasks and dark-acclimated for 30 min with constant agitation at 180 rpm to relax the proton gradient across the thylakoid membrane and allow for full conversion of Zea to Vio. Agitation was used to prevent hypoxia, which is known to induce State 2 in *Chlamydomonas* (Finazzi et al. 2002). 8 to 10 mL of culture was then deposited onto a glass fiber prefilter (Millipore Sigma, MA, USA) using a syringe, the filter was positioned in a leaf clip, and light curves were collected with a Fluorescence Monitoring System FMS 2+ (Hansatech Instruments Ltd, Norfolk, UK) as described previously (Brooks and Niyogi 2011) with gain set to 50 and modulation beam intensity set to 2. The samples were first illuminated with far-red light to induce State 1: samples collected during the light phase were illuminated with far-red light for 10 min, and samples collected during the dark phase were illuminated for 5 min since 77 K fluorescence emission spectra showed that cells were in State 1 in the night. Then, *F_v_/F_m_* was measured using a saturating pulse with an intensity of 45 units and a pulse width of 0.5 s. 5 min treatments of increasing actinic light intensity (50, 200, 500, 1,000, and 1,500 µmol photons m^−2^ s^−1^) were then administered to determine light-adapted $F_{m}^{\prime }$ (using the same saturating pulse settings) and calculate energy dissipation as NPQ = (*F_m_* − $F_{m}^{\prime }$)/$F_{m}^{\prime }$.

### Statistical analysis of physiological data

Experimental replicates refer to independent experiments performed in different weeks. The number of experimental replicates for each physiological measurement is specified in the associated section of the Methods and the figure legends. The following analyses were performed to identify which comparisons were statistically significant using the R statistical programming language. The choice of which statistical test to apply was made independently for each physiological measurement based on three criteria: whether replicate measurements were independent of each other (as opposed to being paired), whether the data could be considered normally distributed, and whether there was equal variance between test groups (homoscedasticity). Experimental replicates were independent of each other for all physiological measurements except for starch content, for which samples were processed and assayed in batches one experimental replicate at a time. The normality of data was determined by evaluation of two inputs: a Shapiro-Wilk’s test for normality (alpha = 0.05), and visual examination of a quantile–quantile (QQ) plot of residuals. For data sets with a normal distribution, equal variance was determined with Levene's test (alpha = 0.05). For physiological measurements with normally distributed data, equal variance, and independent replicates, a two-tailed ANOVA analysis was performed. If the resulting *P*-value was <0.05, Tukey's HSD test was performed as a post-hoc analysis of pairwise comparisons. A multiple testing correction is included in the Tukey HSD test. Pairwise comparisons were considered significant if the adjusted *P*-value was <0.05. For physiological measurements with normally distributed data and independent replicates, but without equal variance, a two-tailed Welch's ANOVA analysis was performed. If the resulting *P*-value was <0.05, a Games-Howell test was performed as a post-hoc analysis of pairwise comparisons. For physiological measurements with data that is not normally distributed, a Kruskal-Wallis test was performed. If the resulting *P*-value was <0.05, a Dunn test was performed as a post-hoc analysis of pairwise comparisons. For physiological measurements with normally distributed, homoscedastic data, but without independent replicates, a repeated measures ANOVA was performed. If the resulting *P*-value was <0.05, a series of Wilcoxon paired tests were performed, followed by multiple testing correction by the Benjamin–Hochberg method. Details about the assumptions, parameters, and results of statistical tests for each physiological measurement are provided in Supplementary Data Set 9.

### Significant differences in gene expression

Differences in mRNA abundance between the three populations (acclimated to LL, ML, and HL) at a given time point were tested for significance with the R package *DESeq2* (v1.44.0). Transcript abundance differences were considered significant if they had a log_2_-transformed fold change > 1 or <−1 and a Benjamini–Hochberg corrected *P*-value < 0.01. The full list of significant changes in mRNA abundance is available in Supplementary Data Set 5. Significant differences between the three populations at a given time are represented as yellow or blue tiles in figures.

Differences in protein abundance between the test populations (acclimated to LL or HL) relative to the control population (acclimated to ML) at a given time point were tested for significance as follows. The average MASIC value for a given protein across the three experimental replicates was calculated, and the log_2_-fold change in the average MASIC value for the test population relative to the ML control population was determined. Then, the log_2_-fold change for a given protein was compared to the log_2_-fold changes for all other proteins using a Z-score analysis. Log_2_-fold changes that were 2 standard deviations above or below the mean log_2_-fold change for the test population relative to the control population (Z-score < −2 or >2) and for which the average MASIC value in both the test and the control population was greater than the limit of quantitation (LOQ) were considered significantly different. The full list of significant changes in protein abundance is available in Supplementary Data Set 5. Significant differences between the three populations at a given time are represented as yellow or blue tiles in figures.

For comparative analyses of differentially expressed genes across the three populations, across time, and across the mRNA and protein levels, modified UpSet plots (Lex et al. 2014) were generated using the R package *ComplexHeatmap* (v2.15.3) (Gu 2022).

### Significant differences in the lipidome

Significant differences in MGDG, DGDG, SQDG, and PG were assessed using a two-way mixed ANOVA using the R package *rstatix* (v0.7.2). Normality was confirmed using boxplots, and equal variance (homoscedasticity) was confirmed using Levene's test (*P* > 0.05 for all groups). Significant effects in accumulation of the four major chloroplast lipid classes were classified as those that had a *P* < 0.05, while significant effects in accumulation of each of the 71 individual lipid species were classified as those that had a Bonferroni *p-adj.* < 0.05. Details about the assumptions, parameters, and results of these ANOVA tests are provided in Supplementary Data Set 9.

### Patterns and clustering of ’omics data

Patterns of gene expression are represented as Z-scores of mean FPKMs for mRNAs and Z-scores of mean MASIC values for proteins across the three experimental replicates. Minimum and maximum FPKM and MASIC values are also shown to demonstrate the magnitude of change over time and across the three populations.

Patterns of lipid content are represented as Z-scores of the mean peak intensity across the three experimental replicates. As similar patterns over light intensity and time were observed across lipid species of the same class (MGDG, DGDG, and SQDG) (Supplementary Fig. S8C), the mean Z-scores and normalized peak intensities were calculated for each lipid class. Mean Z-scores of normalized abundances are used to demonstrate the differences over time and across the three populations, while the minimum and maximum normalized peak intensities are shown to demonstrate the dynamic range for each lipid class.

PCA of the transcriptome, proteome, and lipidome were performed in the R package *PCAtools* (v2.16.0) using the pca function, where the lower 10% of variables were removed according to variance. For the lipidome, PCA was performed separately for the data collected in negative mode and the data collected in positive mode. PCA of the TMT proteomics data revealed that, as expected, the primary data grouping component was TMT plex membership. Yet, PCA of individual plexes indicated high similarity across replicates and the influence of time and light intensity. Therefore, we presented the PCA of the mean protein abundances across the experimental replicates to average out the contribution of TMT plex membership on the data.

The *k*-means clustering analysis was conducted with the R package *stats* (v4.4.0) using the k-means function. First, mean transcript abundances (as FPKMs) and mean protein abundances (as MASIC values) from all nuclear genes were independently Z-score normalized, and then merged. Genes that were undetected in one or both datasets were excluded, leaving 9,821 genes. The k-means function was applied to the resulting data frame with centers = 16 and iter.max = 1,000. Next, genes were arranged by cluster assignment and a heatmap was generated with the heatmaps.2 function from the R package *gplots* (v3.1.3.1).

### Co-expression analysis of photosystem and antenna proteins

PCA was used to probe the co-expression of LHC proteins and the core proteins of the photosystems. The pca function from the R package *PCAtools* (v2.16.0) was applied to the MASIC values measured in the 45 samples (three experimental replicates of three photoacclimated populations at the five timepoints across the diurnal cycle) for the subset of genes pictured (the LHCBMs, LHCBs, PsbA, PsbB, PsbC, and PsbD for Fig. 6E; the LHCBMs, LHCAs, PsaA, PsaB, PsaC, PSAD1, and PSAE1 for Fig. 6G). Co-expression of the proteins was also assessed by calculating the pairwise Pearson correlation coefficient (*r*) for the MASIC values measured in the 45 samples. The cor function from the R package *stats* (v4.4.1) was applied with method = “pearson” and missing values were excluded with use = “pairwise.complete.obs”.

### Rhythmicity analysis

Rhythmic mRNA and protein accumulation were characterized for the LL, ML, and HL populations independently with the R package *DiscoRhythm* (v1.14.0) (Carlucci et al. 2020) using a Cosinor algorithm (Cornelissen 2014), which can tolerate time course data with non-equidistant sampling times. FPKM or MASIC values for the three experimental replicates were double-plotted, and the major expected period length was set to 24 h. The program excluded data with missing or constant values, so rhythmicity was assessed for roughly 13 to 14 × 10^3^ transcripts and roughly 9 × 10^3^ proteins in each of the three populations; full results of the analysis are available as Supplementary Data Set 3. Pearson correlation and PCA were used to confirm that none of the samples was considered an outlier. Periods were detected and fit using a Cosinor model to principal component scores. Oscillations were detected using the Cosinor method, and accumulation was considered “rhythmic” if the *q*-value was < 0.05. To compare the phase of mRNA accumulation to the phase of protein accumulation, only genes whose products were rhythmic at both the mRNA and protein level for a given light intensity were considered (1,004 genes in LL, 2,379 genes in ML, 1,849 genes in HL). To compare the phase of expression in the LL or HL populations relative to ML control population, only genes whose products were rhythmic in both light intensities were considered (roughly 11 to 12× 10^3^ transcripts, roughly 1 to 2× 10^3^ proteins).

### GO enrichment analysis

Previously determined GO term assignments for all genes in *C. reinhardtii* v6.1 genome were downloaded from phytozome.net. Next, genes in each cluster from the *k*-means clustering analysis (Fig. 2G), and the core HL-responsive genes (Fig. 3C) were analyzed for enrichment of GO terms with the R package *clusterProfiler* (v4.12.0) by using the enricher function with pvalueCutoff = 0.05, pAdjustMethod = “BH”. A representative subset of GO terms significantly enriched in the 16 *k*-means clusters is displayed (Fig. 2H and Supplementary Fig. S3), and the full results of the enrichment analysis are available as Supplementary Data Set 4. All significantly enriched GO terms are displayed for the core HL-responsive gene expression changes (Fig. 3C).

### Accession numbers

RNA-Seq raw and analyzed data have been deposited at NCBI Gene Expression Omnibus (GEO) under accession GSE275433 and are publicly available as of the date of publication. Raw proteomics data have been deposited at the ProteomeXchange platform MassIVE under accession MSV000095622 and are publicly available as of the date of publication. Raw lipidomics data have been deposited at the National Metabolomics Data Repository (NMDR) (https://dx.doi.org/10.25582/data.2024-08.3214338/2434308) and are publicly available as of the date of publication.

## Supplementary Material

koaf086_Supplementary_Data

## Data Availability

RNA-Seq raw and analyzed data underlying this article are available in NCBI Gene Expression Omnibus (GEO) under accession GSE275433. Raw proteomics data underlying this article are available in the ProteomeXchange platform MassIVE under accession MSV000095622 (ProteomeXchange accession PXD054947). Raw lipidomics data underlying this article are available in the National Metabolomics Data Repository (NMDR) at https://dx.doi.org/10.25582/data.2024–08.3214338/2434308.

## Supplementary data

The following materials are available in the online version of this article.

**Supplementary Figure S1.** Genome-wide changes in mRNA and protein abundance over the LL, ML, and HL diurnal cycle; related to Fig. 2.

**Supplementary Figure S2.** The transcriptome and proteome are highly reproducible across experimental replicates, even though protein MASIC values were primarily shaped by plex membership in the TMT proteomics data; related to Fig. 2.

**Supplementary Figure S3.** Enrichment of selected GO terms in the 16 clusters in Fig. 2G; related to Fig. 2.

**Supplementary Figure S4.** While most light-responsive changes in transcript abundance are specific to a particular time of day, protein abundance was often constitutively altered in the LL and HL populations; related to Fig. 3.

**Supplementary Figure S5.** Changes in gene expression are most often unique to the mRNA or protein level, even when accounting for a 4 h delay for protein accumulation; related to Fig. 3.

**Supplementary Figure S6.** Core HL-responsive gene expression changes; related to Fig. 3.

**Supplementary Figure S7.** Quantitative image analysis of electron micrographs to describe thylakoid membrane ultrastructure; related to Fig. 5.

**Supplementary Figure S8.** Diurnal photoacclimation leads to stable changes in individual chloroplast lipid species that persist in the dark phase; related to Fig. 5.

**Supplementary Figure S9.** Diurnal photoacclimation results in stable changes to LHC and photosystem protein abundances; related to Fig. 6.

**Supplementary Figure S10.** The Chlamydomonas antenna and cellular pigment profile is dynamic over the diurnal cycle and in response to light intensity; related to Fig. 7.

**Supplementary Data Set 1.** Sample metadata for the transcriptome, proteome, and lipidome; related to Supplementary Data Sets 2 and 7.

**Supplementary Data Set 2.** Genome-wide mRNA abundance (FPKM) and protein abundance (MASIC value) for LL-, ML-, and HL-acclimated *Chlamydomonas* populations across the diurnal cycle in three experimental replicates; related to Fig. 2, Supplementary Figs. S1 to S3.

**Supplementary Data Set 3.**  *DiscoRhythm* analysis of gene expression patterns in the LL-, ML-, and HL-acclimated populations at the mRNA and protein level; related to Fig. 2.

**Supplementary Data Set 4.** Gene ontology enrichment of the 16 clusters of genes; related to Fig. 2 and Supplementary Fig. S3.

**Supplementary Data Set 5.** List of all significant changes in mRNA and protein abundance between the three photoacclimated populations at each timepoint; related to Figs. 3, 4 and 7, Supplementary Figs. S4, S5, and S9.

**Supplementary Data Set 6.** Core HL-responsive gene expression changes; related to Fig. 3 and Supplementary Fig. S6.

**Supplementary Data Set 7.** The lipidome (median-normalized peak intensity) for LL-, ML-, and HL-acclimated *Chlamydomonas* populations across the diurnal cycle in three experimental replicates; related to Fig. 5 and Supplementary Fig. S8.

**Supplementary Data Set 8.** Cellular pigment content (pmol/10^6^ cells) for LL-, ML-, and HL-acclimated *Chlamydomonas* populations across the diurnal cycle in three experimental replicates; related to Figs. 1 and 7 and Supplementary Fig. S10.

**Supplementary Data Set 9.** Statistical analysis of physiological data and lipid abundance; related to Figs. 1, 4, 5, and 7 and Supplementary Fig. S8.

## Dive Curated Terms

The following phenotypic, genotypic, and functional terms are of significance to the work described in this paper:

LCR1 Gramene: Cre09.g399552
LCR1 Araport: Cre09.g399552

LHCBM1 Gramene: Cre01.g066917
LHCBM1 Araport: Cre01.g066917

LHCBM3 Gramene: Cre04.g232104
LHCBM3 Araport: Cre04.g232104

LHCBM5 Gramene: Cre03.g156900
LHCBM5 Araport: Cre03.g156900

LHCBM6 Gramene: Cre06.g285250
LHCBM6 Araport: Cre06.g285250

HLA3 Gramene: Cre02.g097800
HLA3 Araport: Cre02.g097800

CAH4 Gramene: Cre05.g248400
CAH4 Araport: Cre05.g248400

NAR1B Gramene: Cre06.g309000
NAR1B Araport: Cre06.g309000

LHCSR3B Gramene: Cre08.g367400
LHCSR3B Araport: Cre08.g367400

LHCSR3A Gramene: Cre08.g367500
LHCSR3A Araport: Cre08.g367500

RHP1 Gramene: Cre06.g284100
RHP1 Araport: Cre06.g284100

CCM1 Gramene: Cre02.g096300
CCM1 Araport: Cre02.g096300

LCIB1 Gramene: Cre10.g452800
LCIB1 Araport: Cre10.g452800

LCIC1 Gramene: Cre06.g307500
LCIC1 Araport: Cre06.g307500

CAH3 Gramene: Cre09.g415700
CAH3 Araport: Cre09.g415700

CAH6 Gramene: Cre12.g485050
CAH6 Araport: Cre12.g485050

CCP1 Gramene: Cre04.g223300
CCP1 Araport: Cre04.g223300

CCP2 Gramene: Cre04.g222750
CCP2 Araport: Cre04.g222750

LCID1 Gramene: Cre04.g222800
LCID1 Araport: Cre04.g222800

LCIE1 Gramene: Cre04.g223250
LCIE1 Araport: Cre04.g223250

LCI1 Gramene: Cre03.g162800
LCI1 Araport: Cre03.g162800

CAH5 Gramene: Cre05.g248450
CAH5 Araport: Cre05.g248450

PETO1 Gramene: Cre12.g558900
PETO1 Araport: Cre12.g558900

LHCB4 Gramene: Cre17.g720250
LHCB4 Araport: Cre17.g720250

LHCB5 Gramene: Cre16.g673650
LHCB5 Araport: Cre16.g673650

LHCBM2 Gramene: Cre12.g548400
LHCBM2 Araport: Cre12.g548400

LHCBM7 Gramene: Cre12.g548950
LHCBM7 Araport: Cre12.g548950

LHCBM4 Gramene: Cre06.g283950
LHCBM4 Araport: Cre06.g283950

LHCBM8 Gramene: Cre06.g284250
LHCBM8 Araport: Cre06.g284250

LHCBM9 Gramene: Cre06.g284200
LHCBM9 Araport: Cre06.g284200

STT7 Gramene: Cre02.g120250
STT7 Araport: Cre02.g120250

LHCSR1 Gramene: Cre08.g365900
LHCSR1 Araport: Cre08.g365900

PSBS1 Gramene: Cre01.g016600
PSBS1 Araport: Cre01.g016600

PSBS2 Gramene: Cre01.g016750
PSBS2 Araport: Cre01.g016750

CVDE1 Gramene: Cre04.g221550
CVDE1 Araport: Cre04.g221550

ZEP1 Gramene: Cre02.g082550
ZEP1 Araport: Cre02.g082550

ARG7 Gramene: Cre01.g021251
ARG7 Araport: Cre01.g021251
