# Supplementary material for: Too dim, too bright, and just right: Systems analysis of the Chlamydomonas diurnal program under limiting and excess light
Source: Plant Cell. 2025 Apr 19;37(6):koaf086. doi: 10.1093/plcell/koaf086 (PMC12136973; doi:10.1093/plcell/koaf086)
Supplement: koaf086_Supplementary_Data [file koaf086_supplementary_data.zip › TPC-2025-DupuisOjeda_DocumentS1.pdf]

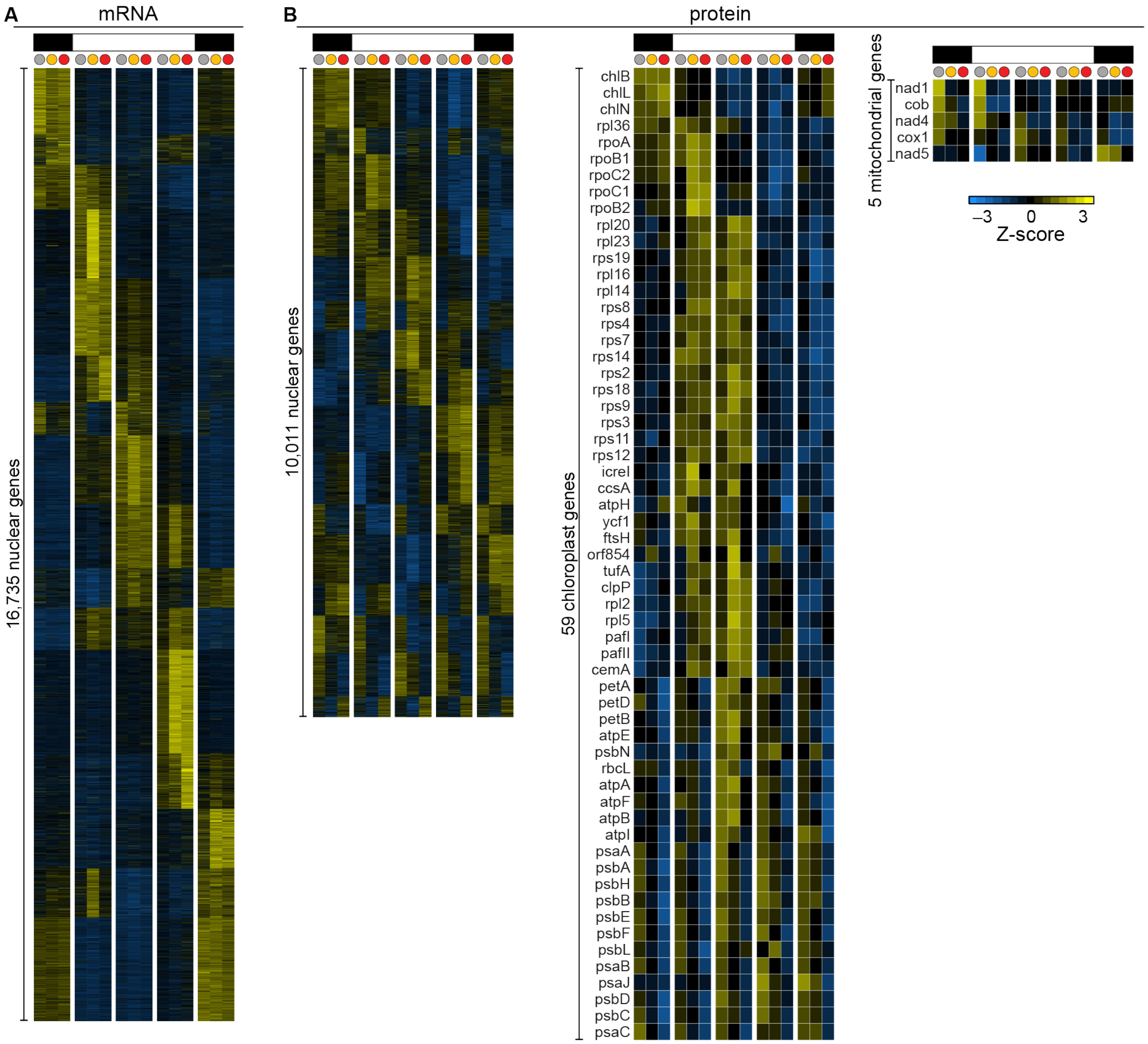

Supplementary Figure S1: Genome-wide changes in mRNA and protein abundance over the low light (LL, grey), moderate light (ML, yellow), and high light (HL, red) diurnal cycle; related to Figure 2.

(A) Normalized abundance of 16,735 nucleus-encoded transcripts detected by RNA-Seq. Normalized values are the Z-score of the mean mRNA abundance (FPKM) of three experimental replicates (n = 3).

(B) Normalized abundance of 10,011 nucleus-encoded proteins, 59 chloroplast-encoded proteins, and 5 mitochondria-encoded proteins detected by TMT proteomics. Normalized values are the Z-score of the mean protein abundance (MASIC value) of three experimental replicates (n = 3).

See also Supplementary Data Set 2.

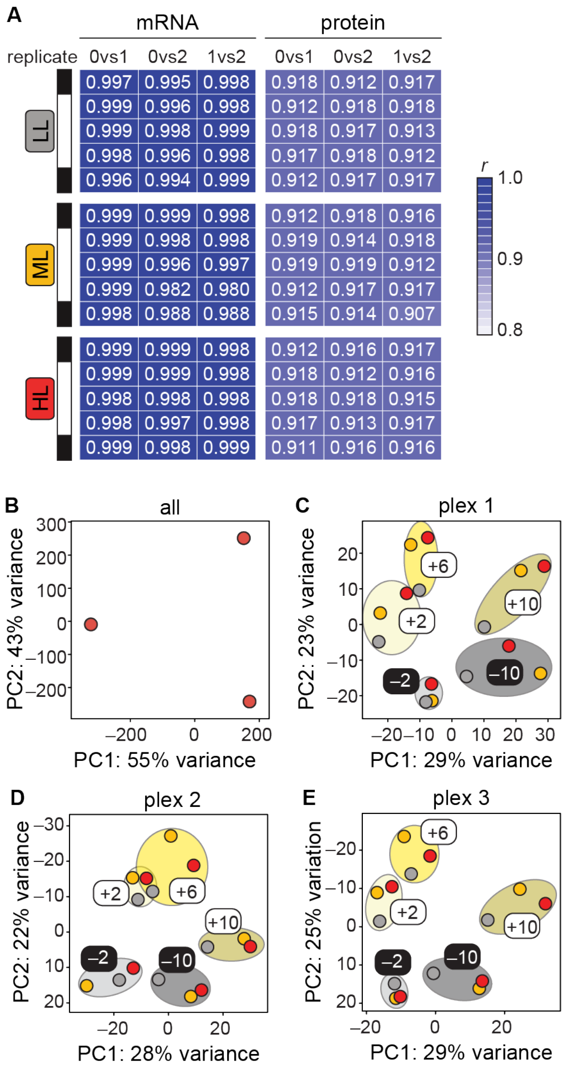

Supplementary Figure S2: The transcriptome and proteome are highly reproducible across experimental replicates, even though protein MASIC values were primarily shaped by plex membership in the TMT proteomics data; related to Figure 2. Gene expression in *Chlamydomonas* populations acclimated to diurnal low light (LL, grey), moderate light (ML, yellow), and high light (HL, red).

(A) Pairwise Pearson correlation coefficients (*r*) of mRNA abundance (FPKM, left) and protein abundance (MASIC value, right) between experimental replicates 0, 1, and 2 of the three photoacclimated populations at the five timepoints across the diurnal cycle.

(B) PCA of protein abundances (MASIC values) genome-wide for all 45 individual samples (three experimental replicates of the three photoacclimated populations sampled at five timepoints). Only three points are apparent, because data points from each of the three randomized sample plexes are completely overlapping. The first two principal components account for 98% of the variation, demonstrating that sample plex drives the variation in protein abundances in the TMT proteomics data.

(C) PCA of protein abundances (MASIC values) genome-wide for samples run in plex 1. The first two principal components account for 52% of the variation.

(D) PCA of protein abundances (MASIC values) genome-wide for samples run in plex 2. The first two principal components account for 50% of the variation.

(E) PCA of protein abundances (MASIC values) genome-wide for samples run in plex 3. The first two principal components account for 54% of the variation.

See also Supplementary Data Set 2.

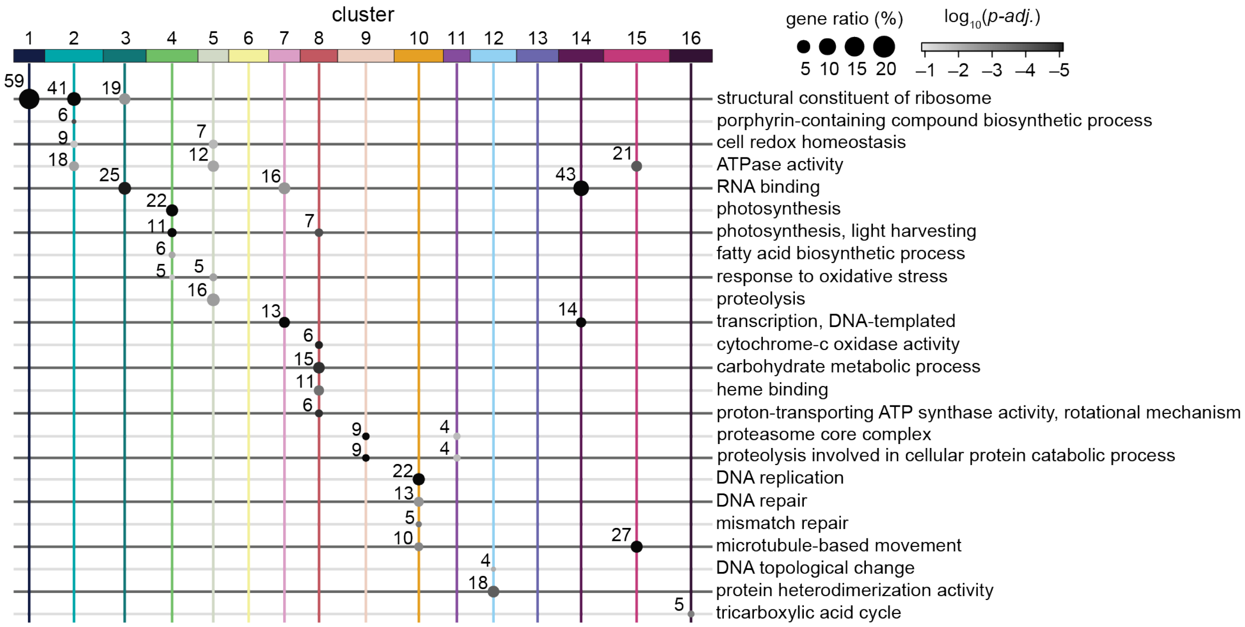

Supplementary Figure S3: Enrichment of selected GO terms in the 16 clusters in Figure 2G; related to Figure 2. Dot size indicates the proportion of genes in the cluster represented by the GO term (relative to the number of genes in the cluster represented by any GO term), dot labels indicate the number of genes, and the shading indicates the $\log_{10}$*p-adj*. The full list of enriched GO terms is available as Supplementary Data Set 4.

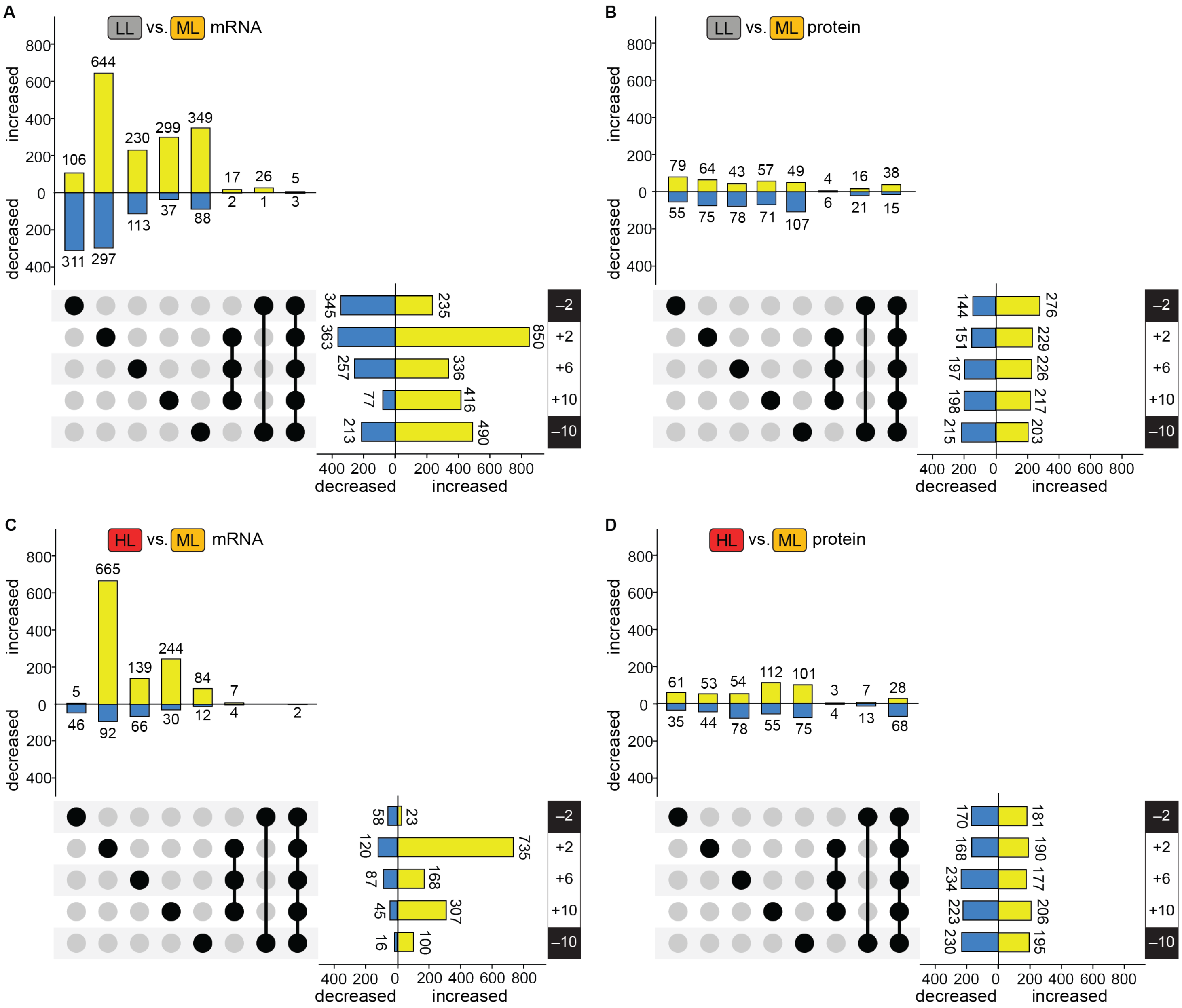
A
LL vs. ML mRNA
increased
decreased
800
600
400
200
0
200
400
106 644 230 299 349 17 26 5
311 297 113 37 88 2 1 3
345 235
363 850
257 336
77 416
213 490
−2
+2
+6
+10
−10
400 200 0 200 400 600 800
decreased increased
B
LL vs. ML protein
79 64 43 57 49 4 16 38
55 75 78 71 107 6 21 15
144 276
151 229
197 226
198 217
215 203
C
HL vs. ML mRNA
5 665 139 244 84 7
46 92 66 30 12 4 2
58 23
120 735
87 168
45 307
16 100
D
HL vs. ML protein
61 53 54 112 101 3 7 28
35 44 78 55 75 4 13 68
170 181
168 190
234 177
223 206
230 195

Supplementary Figure S4: While most light-responsive changes in transcript abundance are specific to a particular time of day, protein abundance was often constitutively altered in the LL and HL populations; related to Figure 3. Column bars represent the number of significant changes unique to each time, those common to the three light-phase timepoints, those common to the two dark-phase timepoints, and those common to all five timepoints (as indicated by the matrix below the columns). Row bars represent the total number of significant changes observed at each timepoint.

(A) Transient and constitutive changes in mRNA abundance in the low light (LL) population relative to the moderate light (ML) population.
(B) Transient and constitutive changes in protein abundance in the LL population relative to the ML population.
(C) Transient and constitutive changes in mRNA abundance in the high light (HL) population relative to the ML population.
(D) Transient and constitutive changes in protein abundance in the HL population relative to the ML population.

See also Supplementary Data Set 5.

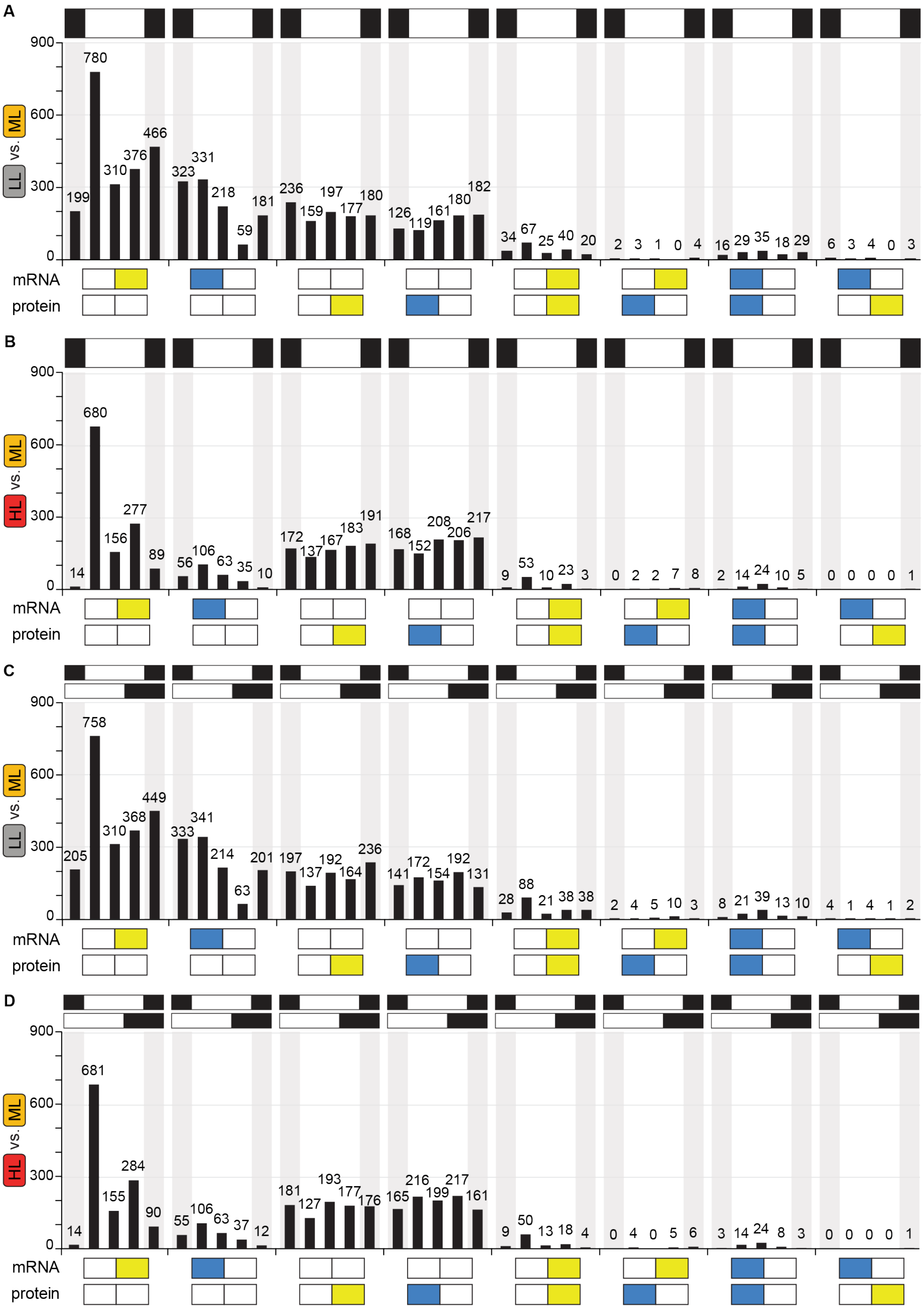
A
900
600
300
0
ML
vs.
LL
199 780 310 376 466
323 331 218 59 181
236 159 197 177 180
126 119 161 180 182
34 67 25 40 20
2 3 1 0 4
16 29 35 18 29
6 3 4 0 3
mRNA
protein
B
900
600
300
0
ML
vs.
HL
14 680 156 277 89
56 106 63 35 10
172 137 167 183 191
168 152 208 206 217
9 53 10 23 3
0 2 2 7 8
2 14 24 10 5
0 0 0 0 1
mRNA
protein
C
900
600
300
0
ML
vs.
LL
205 758 310 368 449
333 341 214 63 201
197 137 192 164 236
141 172 154 192 131
28 88 21 38 38
2 4 5 10 3
8 21 39 13 10
4 1 4 1 2
mRNA
protein
D
900
600
300
0
ML
vs.
HL
14 681 155 284 90
55 106 63 37 12
181 127 193 177 176
165 216 199 217 161
9 50 13 18 4
0 4 0 5 6
3 14 24 8 3
0 0 0 0 1
mRNA
protein

Supplementary Figure S5: Changes in gene expression are most often unique to the mRNA or protein level, even when accounting for a 4 h delay for protein accumulation; related to Figure 3. The schematic x-axis indicates the significant increases (yellow) and decreases (blue) that are unique or shared at mRNA and protein levels.

(A) Comparison of significant mRNA changes to significant protein changes for the low light (LL) population relative to the moderate light (ML) population.

(B) Comparison of significant mRNA changes to significant protein changes for the high light (HL) population relative to the ML population.

(C) Comparison of significant mRNA changes to significant protein changes 4 h later for the LL population relative to the ML population.

(D) Comparison of significant mRNA changes to significant protein changes 4 h later for the HL population relative to the ML population.

See also Supplementary Data Set 5.

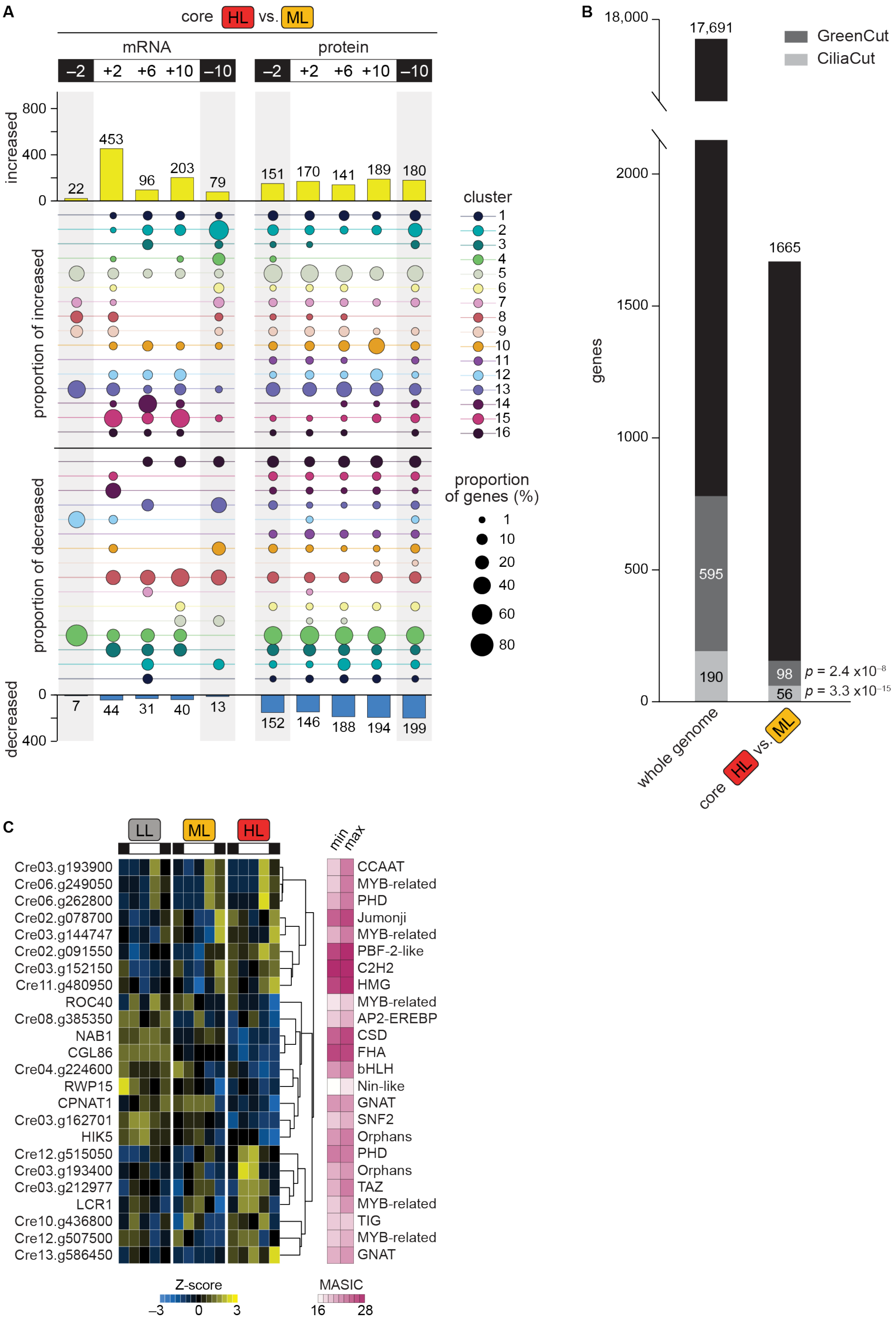
A
core HL vs. ML
mRNA
protein
–2 +2 +6 +10 –10
–2 +2 +6 +10 –10
increased
800
400
0
22
453
96
203
79
151
170
141
189
180
proportion of increased
proportion of decreased
decreased
0
400
7
44
31
40
13
152
146
188
194
199
cluster
1
2
3
4
5
6
7
8
9
10
11
12
13
14
15
16
proportion of genes (%)
1
10
20
40
60
80
B
18,000
2000
1500
1000
500
0
genes
17,691
1665
595
190
98
56
GreenCut
CiliaCut
p = 2.4 x10–8
p = 3.3 x10–15
whole genome
core HL vs. ML
C
LL
ML
HL
min
max
Cre03.g193900
Cre06.g249050
Cre06.g262800
Cre02.g078700
Cre03.g144747
Cre02.g091550
Cre03.g152150
Cre11.g480950
ROC40
Cre08.g385350
NAB1
CGL86
Cre04.g224600
RWP15
CPNAT1
Cre03.g162701
HIK5
Cre12.g515050
Cre03.g193400
Cre03.g212977
LCR1
Cre10.g436800
Cre12.g507500
Cre13.g586450
CCAAT
MYB-related
PHD
Jumonji
MYB-related
PBF-2-like
C2H2
HMG
MYB-related
AP2-EREBP
CSD
FHA
bHLH
Nin-like
GNAT
SNF2
Orphans
PHD
Orphans
TAZ
MYB-related
TIG
MYB-related
GNAT
Z-score
–3
0
3
MASIC
16
28

Supplementary Figure S6: Core HL-responsive gene expression changes; related to Figure 3.

(A) The number of mRNAs significantly increased (top) and decreased (bottom) in abundance at each timepoint in the high light (HL) population relative to the moderate light (ML) population which are not shared with the low light (LL) population (core HL-responsive genes), and the proportion of those genes that belong to the cluster in Figure 2G (relative to the number of the genes that belong to any cluster) represented as dot size (center); the key at right is scaled by the proportion of genes in the genome belonging to each cluster for comparison. A list of these changes is available as Supplementary Data Set 6.

(B) The core HL-responsive genes are significantly enriched for GreenCut genes (genes retained across the green lineages and not in nonphotosynthetic organisms) and CiliaCut genes (genes retained in ciliated organisms and not in non-ciliated organisms)(Merchant et al. 2007). *p* values for enrichment relative to the whole genome are indicated.

(C) Changes in the abundance of known and predicted regulatory proteins curated by Arend et al. 2023 that exhibited HL-specific gene expression changes. Z-scores of mean protein abundances (MASIC value) across three experimental replicates (n = 3) are used to show patterns over time and across the three photoacclimated populations. Proteins were clustered by these patterns. Minimum and maximum FPKM and MASIC values are also shown to demonstrate the dynamic range.

See also Supplementary Data Set 6.

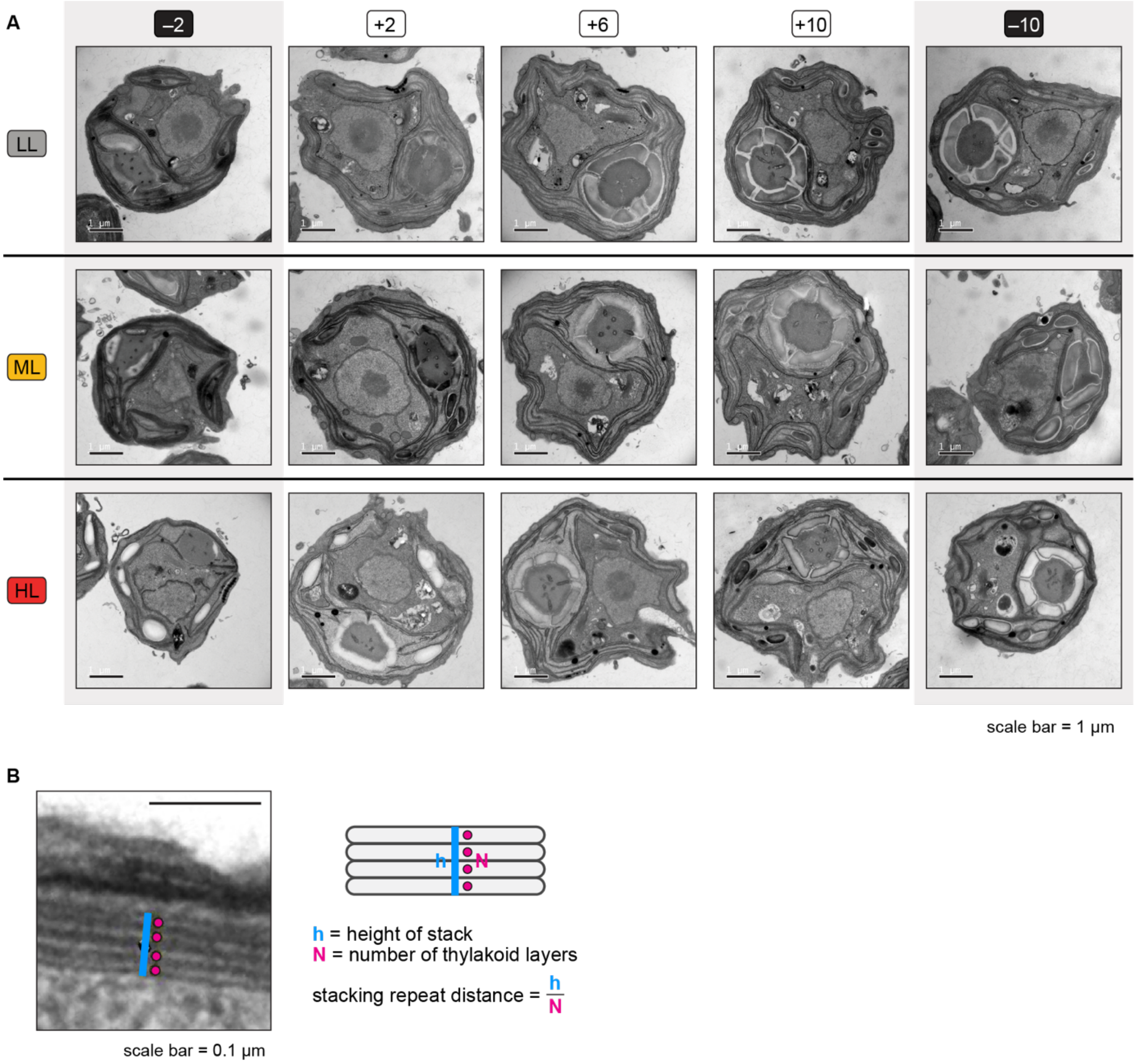

Supplementary Figure S7: Quantitative image analysis of electron micrographs to describe thylakoid membrane ultrastructure; related to Figure 5.

(A) Representative electron micrographs of whole *Chlamydomonas* cells from the 15 cellular states assayed in this study: populations acclimated to diurnal low light (LL, grey), moderate light (ML, yellow), and high light (HL, red) sampled at five timepoints.

(B) Schematic representation of quantitative thylakoid membrane characterization performed using FIJI image analysis software according to Mazur et al. 2021. The membrane height (h) was defined as the distance between the top and bottom layers of the outer thylakoid membranes of the stack. The number of thylakoid membrane layers in the stack (N) was determined, and the stacking repeat distance was calculated by dividing the membrane height by the number of layers.

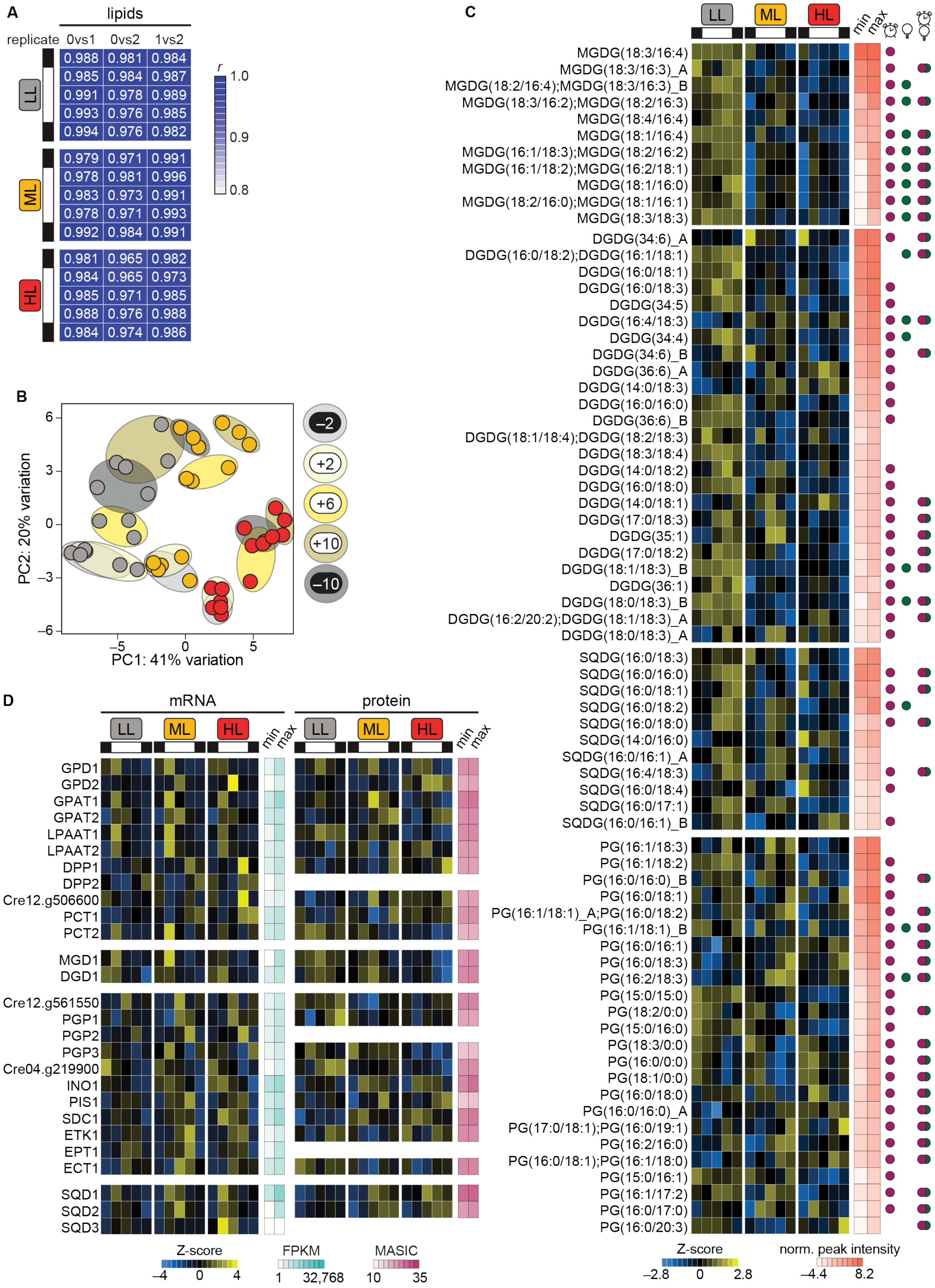

A
lipids
replicate
0vs1
0vs2
1vs2
LL
0.988 0.981 0.984
0.985 0.984 0.987
0.991 0.978 0.989
0.993 0.976 0.985
0.994 0.976 0.982
ML
0.979 0.971 0.991
0.978 0.981 0.996
0.983 0.973 0.991
0.978 0.971 0.993
0.992 0.984 0.991
HL
0.981 0.965 0.982
0.984 0.965 0.973
0.985 0.971 0.985
0.988 0.976 0.988
0.984 0.974 0.986
r
1.0
0.9
0.8
B
PC2: 20% variation
PC1: 41% variation
6
3
0
–3
–6
–5
0
5
–2
+2
+6
+10
–10
C
LL
ML
HL
min
max
MGDG(18:3/16:4)
MGDG(18:3/16:3)_A
MGDG(18:2/16:4);MGDG(18:3/16:3)_B
MGDG(18:3/16:2);MGDG(18:2/16:3)
MGDG(18:4/16:4)
MGDG(18:1/16:4)
MGDG(16:1/18:3);MGDG(18:2/16:2)
MGDG(16:1/18:2);MGDG(16:2/18:1)
MGDG(18:1/16:0)
MGDG(18:2/16:0);MGDG(18:1/16:1)
MGDG(18:3/18:3)
DGDG(34:6)_A
DGDG(16:0/18:2);DGDG(16:1/18:1)
DGDG(16:0/18:1)
DGDG(16:0/18:3)
DGDG(34:5)
DGDG(16:4/18:3)
DGDG(34:4)
DGDG(34:6)_B
DGDG(36:6)_A
DGDG(14:0/18:3)
DGDG(16:0/16:0)
DGDG(36:6)_B
DGDG(18:1/18:4);DGDG(18:2/18:3)
DGDG(18:3/18:4)
DGDG(14:0/18:2)
DGDG(16:0/18:0)
DGDG(14:0/18:1)
DGDG(17:0/18:3)
DGDG(35:1)
DGDG(17:0/18:2)
DGDG(18:1/18:3)_B
DGDG(36:1)
DGDG(18:0/18:3)_B
DGDG(16:2/20:2);DGDG(18:1/18:3)_A
DGDG(18:0/18:3)_A
SQDG(16:0/18:3)
SQDG(16:0/16:0)
SQDG(16:0/18:1)
SQDG(16:0/18:2)
SQDG(16:0/18:0)
SQDG(14:0/16:0)
SQDG(16:0/16:1)_A
SQDG(16:4/18:3)
SQDG(16:0/18:4)
SQDG(16:0/17:1)
SQDG(16:0/16:1)_B
PG(16:1/18:3)
PG(16:1/18:2)
PG(16:0/16:0)_B
PG(16:0/18:1)
PG(16:1/18:1)_A;PG(16:0/18:2)
PG(16:1/18:1)_B
PG(16:0/16:1)
PG(16:0/18:3)
PG(16:2/18:3)
PG(15:0/15:0)
PG(18:2/0:0)
PG(15:0/16:0)
PG(18:3/0:0)
PG(16:0/0:0)
PG(18:1/0:0)
PG(16:0/18:0)
PG(16:0/16:0)_A
PG(17:0/18:1);PG(16:0/19:1)
PG(16:2/16:0)
PG(16:0/18:1);PG(16:1/18:0)
PG(15:0/16:1)
PG(16:1/17:2)
PG(16:0/17:0)
PG(16:0/20:3)
Z-score
–2.8
0
2.8
norm. peak intensity
–4.4
8.2
D
mRNA
protein
LL
ML
HL
min
max
GPD1
GPD2
GPAT1
GPAT2
LPAAT1
LPAAT2
DPP1
DPP2
Cre12.g506600
PCT1
PCT2
MGD1
DGD1
Cre12.g561550
PGP1
PGP2
PGP3
Cre04.g219900
INO1
PIS1
SDC1
ETK1
EPT1
ECT1
SQD1
SQD2
SQD3
Z-score
–4
0
4
FPKM
1
32,768
MASIC
10
35

Supplementary Figure S8: Diurnal photoacclimation leads to stable changes in individual chloroplast lipid species that persist in the dark phase; related to Figure 5. Lipids and proteins in *Chlamydomonas* populations acclimated to diurnal low light (LL, grey), moderate light (ML, yellow), and high light (HL, red).

(A) Pairwise Pearson correlation coefficients (*r*) of lipid abundance (median-normalized peak intensities) between experimental replicates 0, 1, and 2 of the three photoacclimated populations at the five timepoints across the diurnal cycle.

(B) PCA of lipid abundance (median-normalized peak intensities) for lipids detected by LC-ESI-MS/MS in negative ionization mode (PG, PA, PE, and PI species) for three experimental replicates (n = 3); ellipses designate time of day.

(C) Changes in individual MGDG, DGDG, SQDG, and PG lipid species. Z-scores of the mean peak intensities across three experimental replicates (n = 3) are used to show patterns over time and across the three photoacclimated populations. Minimum and maximum peak intensity are also shown to demonstrate the dynamic range. Circles at right indicate a significant effect of time, light intensity, or the interaction between the two by two-way mixed ANOVA (Bonferroni *p-adj* < 0.05).

(D) Changes in mRNAs and proteins involved in membrane glycerolipid metabolism. Z-scores of mean mRNA abundances (FPKM) and Z-scores of mean protein abundances (MASIC value) across three experimental replicates (n = 3) are used to show patterns over time and across the three photoacclimated populations. Minimum and maximum FPKM and MASIC values are also shown to demonstrate the dynamic range.

See also Supplementary Data Sets 7 and 9.

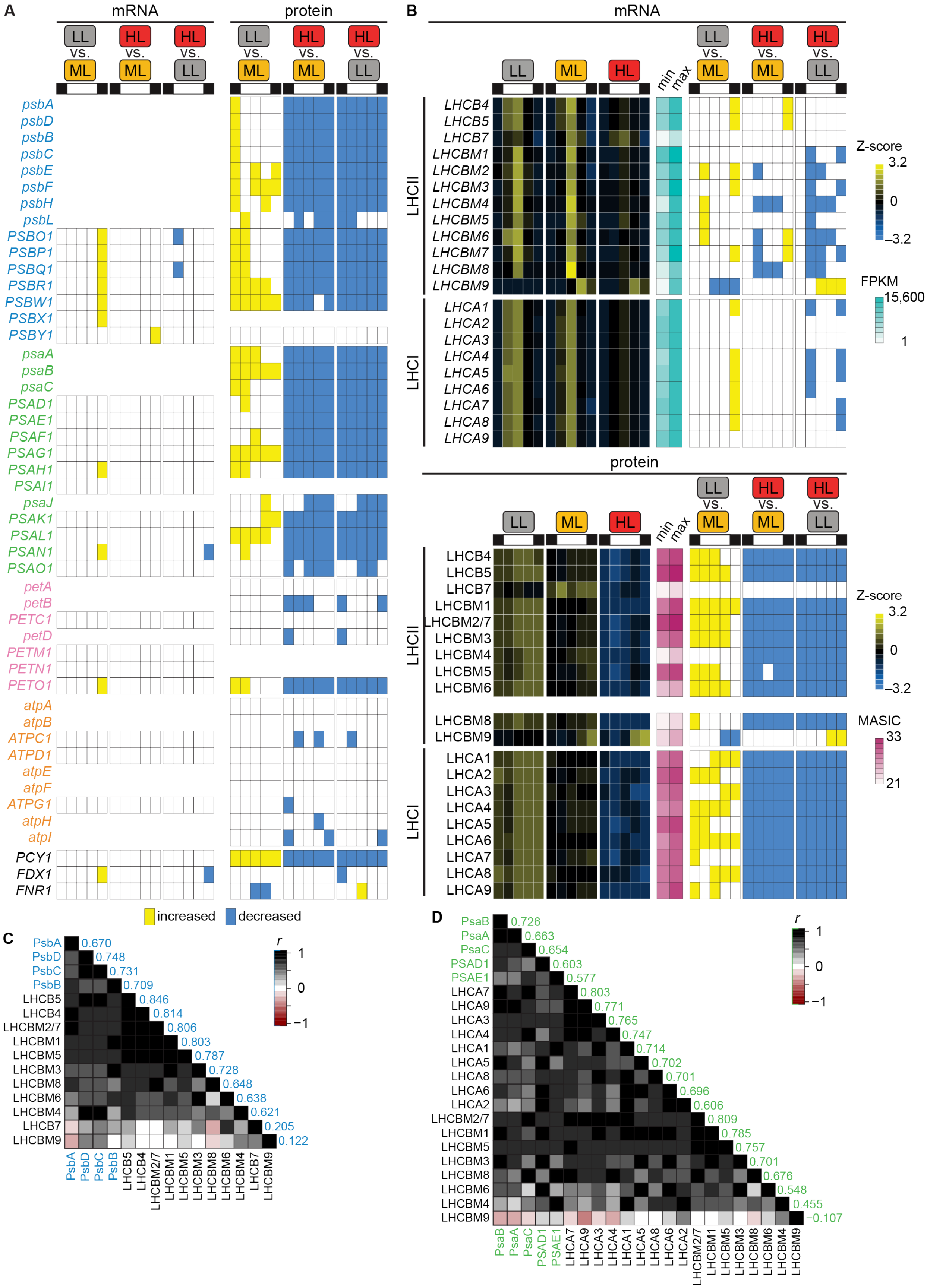

A
mRNA
protein
LL vs. ML
HL vs. ML
HL vs. LL
psbA
psbD
psbB
psbC
psbE
psbF
psbH
psbL
PSBO1
PSBP1
PSBQ1
PSBR1
PSBW1
PSBX1
PSBY1
psaA
psaB
psaC
PSAD1
PSAE1
PSAF1
PSAG1
PSAH1
PSAI1
psaJ
PSAK1
PSAL1
PSAN1
PSAO1
petA
petB
PETC1
petD
PETM1
PETN1
PETO1
atpA
atpB
ATPC1
ATPD1
atpE
atpF
ATPG1
atpH
atpI
PCY1
FDX1
FNR1
increased
decreased
B
mRNA
LL
ML
HL
min
max
LHCII
LHCB4
LHCB5
LHCB7
LHCBM1
LHCBM2
LHCBM3
LHCBM4
LHCBM5
LHCBM6
LHCBM7
LHCBM8
LHCBM9
LHCI
LHCA1
LHCA2
LHCA3
LHCA4
LHCA5
LHCA6
LHCA7
LHCA8
LHCA9
Z-score
3.2
0
–3.2
FPKM
15,600
1
protein
LHCBM2/7
MASIC
33
21
C
PsbA 0.670
PsbD 0.748
PsbC 0.731
PsbB 0.709
LHCB5 0.846
LHCB4 0.814
LHCBM2/7 0.806
LHCBM1 0.803
LHCBM5 0.787
LHCBM3 0.728
LHCBM8 0.648
LHCBM6 0.638
LHCBM4 0.621
LHCB7 0.205
LHCBM9 0.122
r
1
0
–1
D
PsaB 0.726
PsaA 0.663
PsaC 0.654
PSAD1 0.603
PSAE1 0.577
LHCA7 0.803
LHCA9 0.771
LHCA3 0.765
LHCA4 0.747
LHCA1 0.714
LHCA5 0.702
LHCA8 0.701
LHCA6 0.696
LHCA2 0.606
LHCBM2/7 0.809
LHCBM1 0.785
LHCBM5 0.757
LHCBM3 0.701
LHCBM8 0.676
LHCBM6 0.548
LHCBM4 0.455
LHCBM9 –0.107

Supplementary Figure S9: Diurnal photoacclimation results in stable changes to LHC and photosystem protein abundances; related to Figure 6. Gene expression in *Chlamydomonas* populations acclimated to diurnal low light (LL, grey), moderate light (ML, yellow), and high light (HL, red).

(A) Significant increases (yellow) and decreases (blue) in the abundances of mRNAs and proteins of the photosynthetic apparatus across the three populations at each time. Gene names are colored by complex as in Figure 6.

(B) Changes in the expression of LHCs. Individual LHC transcripts and proteins show similar patterns over time in the three populations, except for LHCBM9 and LHCB7. Z-scores of mean mRNA abundances (FPKM) and Z-scores of mean protein abundances (MASIC value) across three experimental replicates (n = 3) are used to show patterns over time and across the three photoacclimated populations. Minimum and maximum FPKM and MASIC values are also shown to demonstrate the dynamic range. Significant increases and decreases between the three populations at a given time are indicated to the right as yellow and blue tiles, respectively.

(C) Coexpression of PSII and LHCII proteins across the 45 samples (three experimental replicates of three photoacclimated populations at the five timepoints across the diurnal cycle), expressed as pairwise Pearson correlation coefficients (*r*). The mean pairwise *r* of each protein relative to the four listed PSII core proteins is shown in blue; pairwise comparisons to self are not included in this mean.

(D) Co-expression of PSI, LHCI, and LHCII proteins across the 45 samples (three experimental replicates of three photoacclimated populations at the five timepoints across the diurnal cycle), expressed as pairwise Pearson correlation coefficients (*r*). The mean pairwise *r* of each protein relative to the five listed PSI core proteins is shown in green; pairwise comparisons to self are not included in this mean.

See also Supplementary Data Set 5.

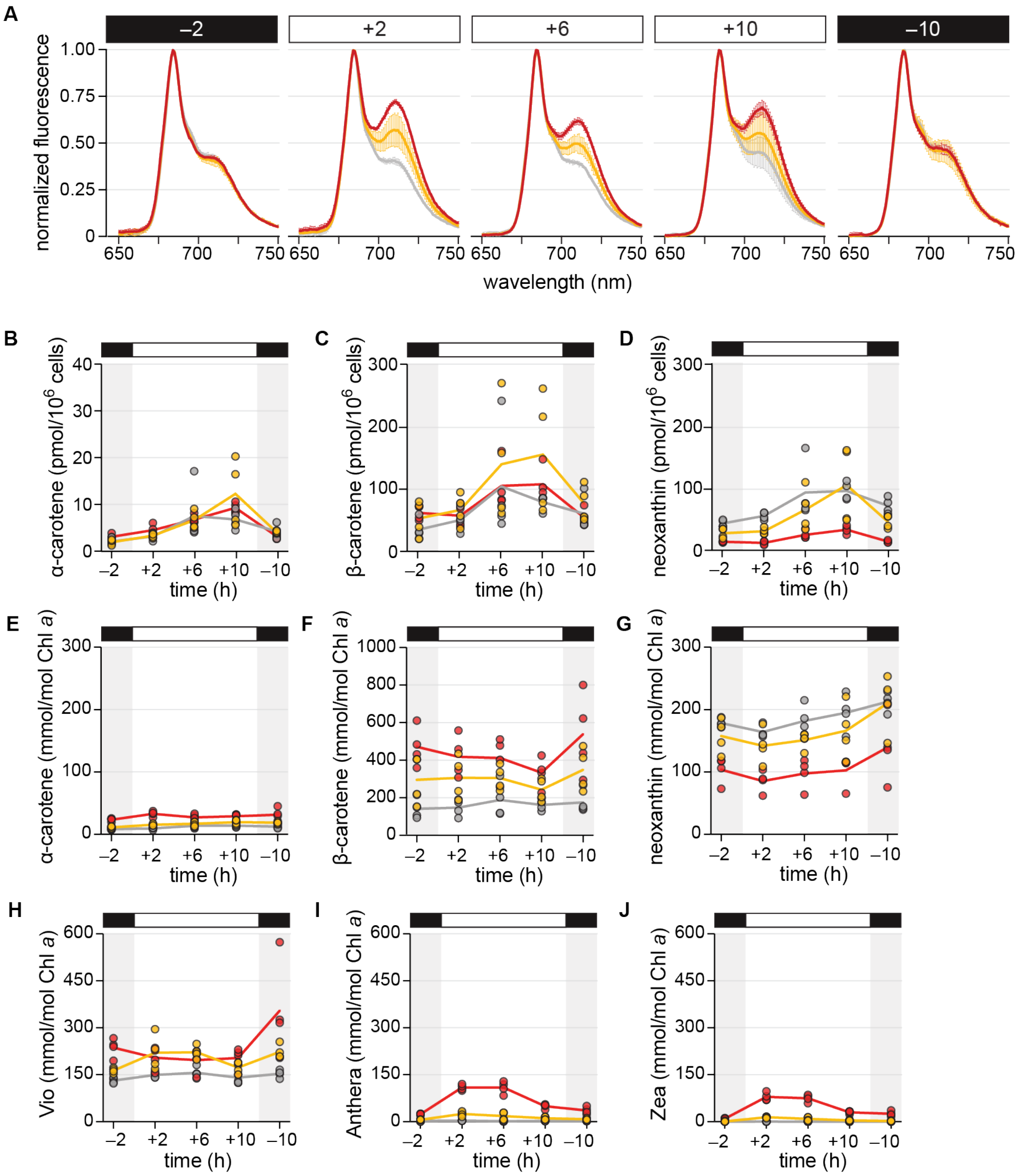

Supplementary Figure S10: The *Chlamydomonas* antenna and cellular pigment profile is dynamic over the diurnal cycle and in response to light intensity; related to Figure 7.

(A) 77 K fluorescence emission spectra of *Chlamydomonas* populations acclimated to diurnal low light (LL, grey), moderate light (ML, yellow), and high light (HL, red). Data are represented as the mean of four experimental replicates (n = 4) with error bars representing the standard deviation from the mean.

(B) Cellular α-carotene concentration measured by HPLC for four experimental replicates (n = 4).

(C) Cellular β-carotene concentration measured by HPLC for four experimental replicates (n = 4).

(D) Cellular neoxanthin concentration measured by HPLC for four experimental replicates (n = 4).

(E) Changes in α-carotene relative to Chl *a* measured by HPLC for four experimental replicates (n = 4).

(F) Changes in β-carotene relative to Chl *a* measured by HPLC for four experimental replicates (n = 4).
(G) Changes in neoxanthin relative to Chl *a* measured by HPLC for four experimental replicates (n = 4).
(H) Changes in Vio relative to Chl *a* measured by HPLC for four experimental replicates (n = 4).
(I) Changes in Anthera relative to Chl *a* measured by HPLC for four experimental replicates (n = 4).
(J) Changes in Zea relative to Chl *a* measured by HPLC for four experimental replicates (n = 4).
